# Migraine and Cardiovascular Risk: A Scoping Review of Vascular Outcomes, Risk Assessment, and Endothelial Dysfunction

**DOI:** 10.3390/life16060900

**Published:** 2026-05-27

**Authors:** Dan Iulian Cuciureanu, Ana-Maria Nădejde, Georgiana-Anca Vulpoi, Cătălina Elena Bistriceanu, Florina Antochi, Adina-Maria Roceanu

**Affiliations:** 1 Neurology Department, Faculty of Medicine, University of Medicine and Pharmacy “Grigore T. Popa”, 16 Universității Street, 700115 Iași, Romania; cuciureanudan@yahoo.com (D.I.C.); vulpoi.anca@yahoo.com (G.-A.V.); catalina_nastac@yahoo.com (C.E.B.); 2 Neurology Department I, “Prof. Dr. N. Oblu” Emergency Clinical Hospital, 2 Ateneului Street, 700309 Iași, Romania; 3 Doctoral School, Faculty of Medicine, University of Medicine and Pharmacy “Grigore T. Popa”, 16 Universității Street, 700115 Iași, Romania; 4 Arcadia Hospitals and Medical Centers, 38 Sarariei Street, 700116 Iași, Romania; 5 Elytis Hospital Hope, 43A Gheorghe Saulescu Street, 700010 Iași, Romania; 6 Neurology Department, University Emergency Hospital, 050098 Bucharest, Romania; flrant@yahoo.com (F.A.); amr2012mar@gmail.com (A.-M.R.)

**Keywords:** migraine, migraine with aura, cardiovascular risk, ischemic stroke, endothelial dysfunction, comorbidities, white matter hyperintensities, vascular biomarkers, scoping review

## Abstract

Migraine affects over one billion individuals worldwide and is increasingly recognized as a systemic disorder with broad cardiovascular comorbidities extending beyond its neurological profile. Despite growing epidemiological evidence, the intersections of migraine with cardiovascular risk stratification, stroke mechanisms, endothelial dysfunction, neuroimaging findings, and neurocognitive profiles remain incompletely mapped. Following the PRISMA-ScR framework, we searched PubMed/MEDLINE for recent (2015–2025) English-language human studies with large sample sizes (≥400) examining cardiovascular outcomes, vascular biomarkers, neuroimaging, or endothelial function in adult migraine populations. The protocol was preregistered on OSF. Forty-three studies encompassing more than 1,500,000 participants across seven thematic domains were included. Migraine with aura was consistently associated with increased ischemic stroke risk in a non-atherosclerotic pattern, with evidence suggesting potential cardioembolic mechanisms including patent foramen ovale and atrial fibrillation. Active episodic migraine paradoxically showed inverse associations with traditional cardiovascular risk scores, while inflammatory biomarkers such as high-sensitivity C-reactive protein and fibrinogen remained interictally elevated, and white matter hyperintensity burden was substantially higher than controls, though available evidence did not indicate increased dementia risk. Cardiovascular disease may represent a clinically relevant comorbidity of migraine, warranting integrated multidisciplinary management. Future studies should prioritize sex-stratified longitudinal designs with aura-specific risk modeling and cerebral endothelial assessment.

## 1. Introduction

Migraine is characterized as a disabling primary headache disorder, typically involving recurrent, moderate to severe unilateral pulsatile headaches. Migraine affects approximately 1.16 billion individuals worldwide as of 2021, representing a 58.15% increase in prevalence cases since 1990, with projections indicating a continued rise in both male and adolescent populations through 2050. The condition disproportionately affects women, with females exhibiting higher absolute rates of migraine incidence and prevalence than male individuals, though the rate of increase has been more rapid in male individuals, and the condition is associated with a 58.27% increase in DALYs globally (disability-adjusted life years). This is consistent with migraine being one of the top causes of disability worldwide [1]. Beyond its neurological manifestations, migraine is increasingly recognized as a complex systemic disorder with a broad network of comorbidities, including cardiovascular (CV), metabolic, and inflammatory conditions, whose shared biological pathways have important implications for internal medicine practice.

The purpose of this scoping review was to systematically map the current evidence on the association between migraine and CV risk across seven thematic domains (cardiovascular risk factors (CVRFS) and scores, stroke and cerebrovascular outcomes, structural and functional vascular assessment, inflammatory and circulating biomarkers, neuroimaging findings, genetic and molecular biomarkers, and neurocognitive profiles) in adult populations. Gender-specific risk profiles were examined throughout, with particular attention to the role of migraine with aura (MA) as a distinct vascular phenotype.

Epidemiological evidence consistently suggests approximately doubled ischemic stroke (IS) risk in migraineurs compared to non-migraineurs [2], predominantly driven by MA rather than migraine without aura (MO) [3], and particularly pronounced in young adults and women [4]. Risk is further amplified in women under 45 with MA who smoke or use oral contraceptives [2,3].

The underlying mechanisms point to non-atherosclerotic pathways. Nathan et al. [5] identified cortical spreading depression as central to MA pathophysiology, proposing mechanistic links including endothelial dysfunction, platelet and coagulation abnormalities, inflammation, and paradoxical embolism. Dzator et al. [6] demonstrated increased arterial stiffness and impaired cerebral vasodilator capacity, predominantly in the posterior circulation, suggesting vascular dysfunction contributes to stroke risk independently of aura status. Davis et al. [7] cautioned, however, that Patent Foramen Ovale (PFO)–stroke associations are weaker in population-based than case–control designs, indicating potential selection bias in earlier estimates.

Endothelial dysfunction represents a critical mechanistic link between migraine and CV risk. Disruption of nitric oxide (NO) bioavailability, calcitonin gene-related peptide (CGRP) signaling, and endothelin 1 (ET-1) balance may predispose migraineurs to vascular events [8,9]. Cortical spreading depression (CSD) triggers trigeminovascular activation with CGRP and substance P release, inducing vasodilation and sterile inflammation [10], while reduced circulating endothelial progenitor cells (EPCs) in MA suggest impaired vascular repair capacity [8,11]. Despite elevated endothelial activation markers, functional flow-mediated dilation (FMD) assessments and structural arterial stiffness measures remain inconsistent [8].

In migraine, elevation of C-reactive protein (CRP) may be caused by oxidative stress, leukocyte activation, inflammatory dilation of blood vessels and inflammatory cytokines, which are increased during acute attacks and between attacks of migraine. Also, specific abnormalities in inflammatory marker levels in the systemic circulation have been observed, including elevated CRP levels. High-sensitivity C-reactive protein (hs-CRP) is a marker of inflammation that may predict subclinical atherosclerosis. Also, the prominent vascular component of migraine has raised growing interest in its potential links with stroke and cognitive decline. Migraine is also a common headache disorder that has been linked to cerebral white matter hyperintensities (WMHs) on MRI, which may resemble infarct-like lesions. Although the underlying pathophysiology of WMHs remains unclear, their development in migraine may be related to the cumulative impact of recurrent intracerebral hemodynamic changes [12]. Because stroke, cerebral WMHs, silent infarcts, and brain volumetric changes are all associated with an increased risk of cognitive impairment, migraines have been proposed as a possible risk factor for cognitive decline and dementia [13].

Another important aspect addressed in this review is that migraine is frequently associated with a positive family history, although its genetic basis remains incompletely understood. Studies investigating the relationship between candidate genetic variants and migraine susceptibility have yielded heterogeneous and sometimes conflicting results. We discuss two relevant studies that examined the potential association between functional single-nucleotide polymorphisms (SNPs) in the neuronal nitric oxide synthase gene (NOS) and migraine risk.

Understanding these CV comorbidities through a multidisciplinary lens as integrating neurology, internal medicine, and vascular biology is essential for improving risk stratification and personalized care in migraine patients. Despite growing evidence, significant gaps persist, such as the relative contributions of aura status, attack frequency, and chronicity to vascular risk, which remain incompletely characterized. Whether endothelial dysfunction is systemic or cerebral-localized has not been comprehensively mapped. Risk stratification tools specific to migraineurs are lacking, and the clinical utility of vascular biomarkers remains uncertain.

This scoping review was, therefore, conducted to systematically map evidence across seven thematic domains: CVRFs and scores, stroke and cerebrovascular outcomes, structural and functional vascular assessment, inflammatory and circulating biomarkers, neuroimaging findings (WMHs), genetic and molecular biomarkers (NOS gene), and neurocognitive profiles.

The current review sought to answer the following primary question: What is the current state of evidence on the association between migraine and CV risk, encompassing vascular outcomes, endothelial dysfunction, and CV risk assessment in adult populations (≥18 years), as published in English-language human clinical studies between 2015 and 2025?

The scoping review was further guided by the following secondary research questions:

- What is the association between migraine and CVRFs and risk scores in adult populations?
- What is the relationship between migraine and stroke risk and cerebrovascular outcomes?
- What structural and functional vascular abnormalities (carotid intima-media thickness—cIMT, pulse wave velocity—PWV, FMD, coronary artery calcification—CAC) are documented in migraine patients?
- Which inflammatory and circulating biomarkers (von Willebrand factor—vWF, hs-CRP, fibrinogen) are associated with endothelial dysfunction in migraine?
- What radiological and neuroimaging findings (WMH) are documented in adult migraine patients?
- What genetic and molecular variants (NOS1, NOS3, low-density lipoprotein receptor-related protein 1—LRP1) are associated with migraine and vascular risk?
- What neurocognitive profiles and dementia risk are documented in adult migraine patients?

## 2. Methodology

### 2.1. Protocol and Methodological Framework

This scoping review follows the methodological framework established by Arksey & O’Malley [14], as refined by Levac et al. [15], and adheres to the Preferred Reporting Items for Systematic Reviews and Meta-Analyses extension for Scoping Reviews (PRISMA-ScR) reporting guidelines [16]. This methodology was specifically selected to map the complex intersection of migraine and CV risk, a field characterized by significant clinical and methodological heterogeneity. Unlike traditional systematic reviews that typically address narrow clinical questions, a scoping approach is uniquely suited to synthesize evidence across diverse research domains, including CV risk assessment, stroke risk, endothelial dysfunction, inflammatory and genetic biomarkers (NOS gene), structural and functional vascular assessments, neuroimaging findings (WMHs), and neurocognitive profiles. This framework ensures a rigorous synthesis of how research was conducted across multiple categories, allowing clarification of key concepts and identification of critical knowledge gaps that a more restrictive review might overlook.

We registered the protocol and search strategy for this scoping review on the Open Science Framework (OSF) [DOI: 10.17605/OSF.IO/YV95Z].

### 2.2. Conceptual Themes Explored in the Synthesis

To structure the evidence synthesis, seven conceptual themes were identified a priori, reflecting the principal domains at the intersection of migraine and cardiovascular risk. These themes guided the selection, mapping, and interpretation of the included literature, without presupposing specific directional outcomes.

T1—“Healthy Vascular System” Paradox. This review examined whether migraine demonstrates an inverse or neutral association with traditional CVRFs in general populations, and whether this relationship shifts with disease chronicity and age. Evidence was assessed for differential associations between chronic migraine, MA, and cardiovascular outcomes including hypertension, atherosclerotic cardiovascular disease, and major cardiovascular events, with attention to gender-specific profiles.

T2—Inflammatory Biomarkers and Endothelial Activation. The synthesis explored whether inflammatory and circulating biomarkers (hs-CRP, fibrinogen) are elevated in migraineurs compared to controls, even during interictal periods. Attention was given to whether these elevations are more pronounced in MA, potentially reflecting a chronic systemic proinflammatory and prothrombotic state.

T3—Migraine-Related Stroke is Non-Atherosclerotic. Evidence was assessed for the hypothesis that MA-associated ischemic stroke occurs primarily through cardioembolic rather than atherosclerotic pathways. The synthesis examined associations between MA, PFO, large artery atherosclerosis, and whether late-onset MA (≥50 years) confers elevated long-term stroke risk.

T4—Absence of Macrovascular Structural Damage; Functional Hemodynamic Impairment. This review examined whether migraine shows independent associations with markers of macrovascular structural damage, including PWV, CAC, intracranial atherosclerosis, and retinal microvascular caliber. The synthesis explored the distinction between structural vascular integrity and functional hemodynamic alterations in migraine pathophysiology.

T5—White Matter Hyperintensities as Consistent Neuroimaging Finding. The synthesis assessed whether migraineurs demonstrate higher WMH burden compared to controls. Evidence was examined regarding whether WMH progression is driven primarily by age and traditional CVRFs versus migraine-specific mechanisms, and the clinical utility of routine neuroimaging for migraine subtype differentiation.

T6—Genetic Variants in Nitric Oxide Pathway. This review explored the role of polymorphisms in NOS1 and NOS3 genes in migraine susceptibility, and whether variants such as LRP1 highlight vascular wall integrity as a genetically determined predisposition. The synthesis examined whether nitric oxide pathway genetics represent primary drivers of migraine or secondary modulators in specific subgroups.

T7—“Migraine-Cognition Paradox”. Evidence was assessed for the relationship between migraine, structural brain lesions, and cognitive outcomes including dementia risk. The synthesis explored whether migraineurs, particularly those with MA, demonstrate preserved or enhanced cognitive performance despite radiological findings, potentially reflecting neuroplastic or compensatory mechanisms whereby vascular and structural risks do not translate into neurodegenerative outcomes.

### 2.3. Eligibility Criteria

Eligibility criteria were defined using the population, concept, context (PCC) framework.

#### 2.3.1. Population

We included adults aged 18 years or older diagnosed with any migraine subtype (episodic migraine, chronic migraine, MA, MO) according to International Classification of Headache Disorders (ICHD) [17] criteria or established clinical diagnostic criteria. To maintain focus on primary CV risk in non-pregnant adults, we excluded pediatric populations (with one exception for early-onset vascular data), pregnancy-related conditions (e.g., preeclampsia), and secondary vascular disorders such as cerebral autosomal dominant arteriopathy with subcortical infarcts and leukoencephalopathy (CADASIL).

#### 2.3.2. Concept

This review encompassed vascular outcomes, endothelial function, CV risk markers, stroke risk, neuroimaging findings, and neurocognitive profiles associated with migraine.

#### 2.3.3. Context

We included all primary study designs: cross-sectional studies, case–control studies, cohort studies, and randomized controlled trials. We excluded animal and in vitro models, case reports with fewer than 10 participants, and secondary literature (reviews, editorials, commentaries). This approach ensures the synthesis reflects robust human clinical evidence across the seven thematic categories of interest.

#### 2.3.4. Temporal and Language Restrictions

The search was restricted to English-language studies published between 2015 and 2025. This timeframe was strategically chosen to build upon the review by Butt et al. [8]. By focusing on the last decade, this scoping review captures modern advancements in radiological findings, genetic biomarkers, and novel vascular assessment technologies that have emerged since the 2015 baseline, providing an up-to-date map of the field’s progression.

### 2.4. Information Sources and Search Strategy

#### 2.4.1. Database and Rationale

Although complementary databases such as Embase, Scopus, and the Cochrane Library offer broader coverage in some domains, the literature search was conducted exclusively within PubMed/MEDLINE, with temporal restriction (2015–2025) and language filter (English only) applied. According to the National Library of Medicine (2024), this decision was based on PubMed/MEDLINE’s comprehensive indexing of peer-reviewed cardiovascular, neurological, and vascular medicine literature, encompassing over 36 million citations across MEDLINE, PubMed Central, and publisher-supplied records. The search strategy was iteratively developed using a Boolean combination of three primary blocks applied to title and abstract fields, prioritizing free-text terms over Medical Subject Headings (MeSH) to maximize sensitivity and capture emerging terminology not yet fully represented in controlled vocabularies. In accordance with the PRISMA-ScR framework, which requires transparent reporting of information sources and search methods without prescribing a minimum number of databases [16], this approach reflects a deliberate balance between operational feasibility and the broad, mapping-oriented objectives of scoping review methodology. The single-database approach is acknowledged as a limitation (see Section 4.10).

#### 2.4.2. Search Construction

This search strategy was iteratively developed to balance sensitivity and specificity, using a Boolean combination of three primary blocks. To capture the most recent advancements and emerging terminology, free-text terms were utilized in the title and abstract fields rather than relying solely on Medical Subject Headings (MeSH) terms.

Block 1 (Migraine): To ensure migraine was the central focus of retrieved studies, ten specific migraine-related terms (e.g., “chronic migraine”, “migraine disorders”, “migraine with aura”, “migraine without aura”) were restricted to the article title field.

Block 2 (Vascular and Endothelial): To capture the breadth of vascular and endothelial outcomes, 17 thematic sub-blocks were searched across titles and abstracts. These encompassed the following:

- Functional assessments: FMD, cerebrovascular reactivity, EPCs;
- Structural assessments: cIMT, PWV, CAC;
- Circulating biomarkers: vWF, ET-1, vascular endothelial growth factor (VEGF), asymmetric dimethylarginine (ADMA), endothelial nitric oxide synthase (eNOS), adhesion molecules (ICAM-1, VCAM-1, E-selectin), pentraxin-3 (PTX3);
- Vascular pathology: atherosclerosis, arterial stiffness, endothelial dysfunction;
- Neuroimaging: WMH, cerebrovascular lesions;
- Genetic markers: NOS variants, LRP1.

Block 3 (Exclusion Filters): Exclusion terms were applied to remove studies focused on pregnancy-related conditions, pediatric populations, animal models, and specific secondary vascular conditions (e.g., CADASIL).

Applied Filters: The search was limited to studies published between 2015 and 2025, English language, abstract availability, human species, and adult populations only.

The complete search string is provided in Supplementary File S2, Table S8.

Temporal Parameters and Yield: The search covered the decade from January 2015 to December 2025, capturing the evolution of the field since the 2015 Butt et al. review [8]. The initial query yielded 161 records for title and abstract screening.

### 2.5. Study Selection Process

#### 2.5.1. Screening Procedure

Titles and abstracts of all retrieved records were independently screened by two reviewers (A.-M.N. and G.-A.V.) against the predefined eligibility criteria. Full-text articles were subsequently assessed for final inclusion. Disagreements at any stage were resolved through discussion, with arbitration by a third reviewer (C.E.B.) when consensus could not be reached.

#### 2.5.2. Documentation

The study selection process was documented in accordance with PRISMA-ScR guidelines. A PRISMA flow diagram was designed to illustrate the number of records identified, screened, assessed for eligibility, and included in the final synthesis. Studies excluded at the full-text stage were recorded with reasons for exclusion.

A minimum sample size threshold of N ≥ 400 participants was applied as a pragmatic eligibility refinement to ensure a manageable and high-quality scope for this scoping review. The ≥400 participant threshold was implemented as a strict proxy for statistical power and internal consistency, designed to reduce the well-documented heterogeneity and effect-size inflation associated with small-sample clinical studies in cardiovascular and neurological research. This volumetric filter ensures comparability across the seven thematic domains mapped in this review by anchoring the evidence base to large-scale observational cohorts with adequate statistical robustness. We explicitly acknowledge that this threshold consequently omits smaller mechanistic, proof-of-concept imaging, and exploratory biomarker studies that may hold significant descriptive value independent of sample size. A deliberate operational boundary is further discussed in the Limitations section. Such refinements are consistent with established scoping review methodologies, which allow for iterative inclusion criteria to align the evidence synthesis with the research objectives and the practical necessity of focusing on high-precision data.

The search strategy was strictly tailored to capture biomarkers of systemic endothelial cell activation and vascular stress. Consequently, studies evaluating classic hematological thrombophilia profiles (e.g., protein C, protein S, antithrombin III, factor V Leiden, APCR, lupus anticoagulant, anticardiolipin, and anti-β2-glycoprotein 1 antibodies) fell outside the primary scope of this vascular-focused search string and were not actively retrieved, as these terms were not included among the predefined vascular search blocks. This deliberate boundary reflects the distinct pathophysiological framework of our review, which focuses on chronic, low-grade endothelial dysfunction and systemic vascular stress rather than acute coagulation cascade disorders or inherited hypercoagulable states.

The OSF protocol was registered retrospectively, following completion of the search strategy but prior to data extraction and synthesis, to ensure transparency of the review process.

### 2.6. Data Charting and Extraction

A standardized data charting form was developed and piloted prior to extraction. For each included study, the following data were extracted: author and year of publication; study design; sample size (migraine group and control group); migraine subtype(s); assessment method (CV or vascular outcome(s) assessed, including the specific tool, biomarker, or imaging modality used); main findings with exact numbers (effect sizes, confidence intervals, and *p*-values where reported); and interpretation (clinical significance of findings in the context of migraine-associated vascular risk).

Data were extracted by one reviewer (A.-M.N.) and verified by a second reviewer (G.-A.V.). Extracted data were organized into seven thematic categories corresponding to the secondary research questions: (1) CV risk assessment, (2) stroke risk and cerebrovascular outcomes, (3) structural and functional vascular assessment, (4) inflammatory and circulating biomarkers, (5) radiological and neuroimaging findings, (6) genetic and molecular biomarkers, and (7) neurocognitive profiles.

### 2.7. Synthesis Approach

#### 2.7.1. Narrative Synthesis

Given the significant clinical and methodological heterogeneity across included studies, encompassing diverse study designs, migraine subtypes, outcome measures, and analytical approaches, a quantitative meta-analysis was not performed. Instead, a structured narrative synthesis was conducted for each of the seven thematic categories. Within each category, findings were summarized descriptively, with attention to effect size direction and magnitude, consistency of findings across studies, and subgroup differences by migraine subtype (particularly MA versus MO), sex, and age.

#### 2.7.2. Methodological Decisions

Studies were not formally appraised for methodological quality or risk of bias, consistent with the scoping review methodology, which prioritizes breadth of evidence mapping over quality assessment [14,15]. However, study design, sample size, and analytical approach were considered when interpreting and contextualizing findings.

#### 2.7.3. Contingency Plan

If narrative synthesis within a thematic category was not possible due to insufficient extractable data, findings were reported descriptively on a study-by-study basis, with explicit acknowledgment of the limited evidence base.

#### 2.7.4. Reporting

The final synthesis was reported in accordance with PRISMA-ScR guidelines [16]. Findings for each thematic category are presented in structured evidence tables followed by a narrative synthesis that contextualizes results within the broader literature.

The study selection process is illustrated in the PRISMA flow diagram presented in Section 3 (Results). Full evidence tables are provided in Supplementary File S1, Tables S1–S7; the PRISMA-ScR checklist is available in Supplementary File S2, Table S8.

## 3. Results

The systematic search of PubMed/MEDLINE yielded 161 records, of which 118 were excluded following title and abstract screening for relevance and preliminary eligibility. The remaining studies underwent a two-stage evaluation: first, assessment of the minimum sample size requirement of ≥400 participants, and subsequently, comprehensive full-text review against all predefined inclusion criteria.

**Figure 1:**
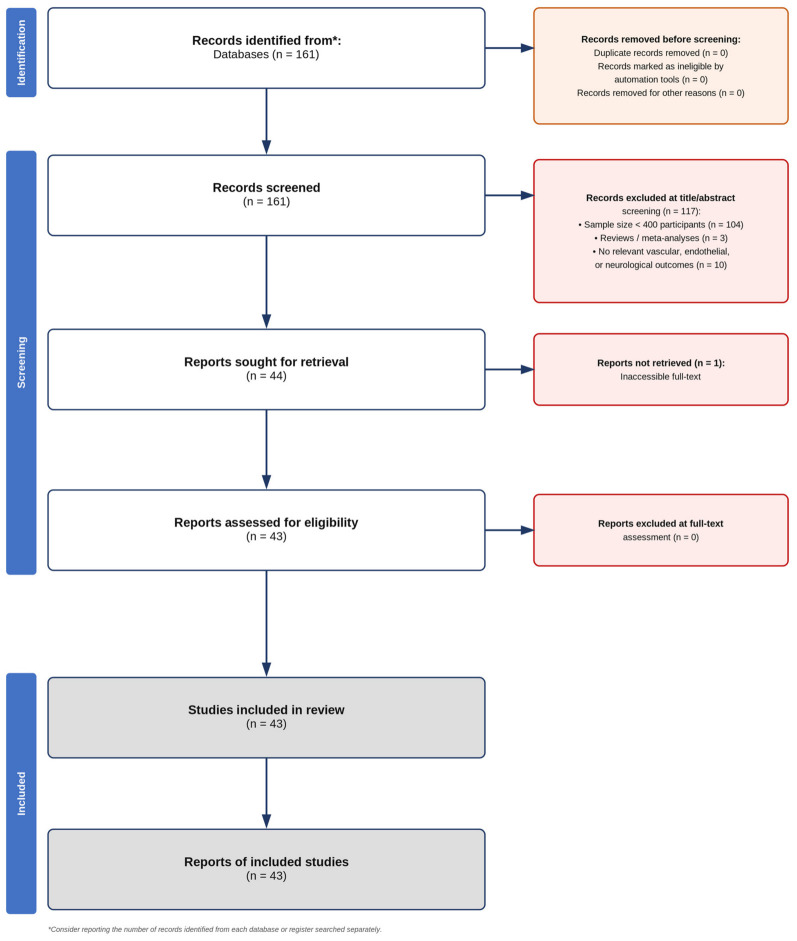
PRISMA flowchart indicating the publication screening and selection process.

Following this process, 117 studies were excluded for the following reasons: 104 studies did not meet the minimum sample size threshold of 400 participants (including control groups); 3 studies were identified as review articles or meta-analyses; and 10 studies, despite meeting the sample size requirement, did not fulfil other inclusion criteria (e.g., absence of relevant CVRFs, vascular outcomes, neurological outcomes or endothelial dysfunction markers as primary outcomes). Of the 44 studies deemed eligible, one was subsequently excluded due to inability to retrieve the full-text article. The final dataset comprised 43 studies meeting all inclusion criteria. The study selection process is illustrated in the PRISMA flow diagram (Figure 1).

The 43 included studies encompassed a total of over 1,500,000 participants across diverse populations and geographic regions. Studies were categorized into seven thematic domains based on their primary outcomes: CV risk assessment (*n* = 14), stroke risk and outcomes (*n* = 13), structural and functional vascular assessment (*n* = 8), inflammatory and circulating biomarkers (*n* = 2), radiological and neuroimaging findings (*n* = 2), genetic and molecular biomarkers (*n* = 3), and neurocognitive profiles (*n* = 2); one study contributed data to two categories. Study designs included prospective cohorts (*n* = 15), retrospective cohorts (*n* = 10), cross-sectional studies (*n* = 10), and case–control studies (*n* = 6), with each thematic category being discussed below, and findings drawn exclusively from the included studies and interpreted based on the extracted data. Sample sizes ranged from 415 to ~1.19 M participants, with all studies meeting the minimum threshold of 400 participants. Most studies (*n* = 35, ~80%) distinguished between MA and MO, while the remaining studies examined migraine as a unified diagnostic category. Study characteristics and main findings are summarized in Supplementary File S1, Tables S1–S7.

### 3.1. Cardiovascular Risk Assessment

Table 1 presents the CV risk assessment findings from 14 included studies [18,19,20,21,22,23,24,25,26,27,28,29,30,31], summarizing study designs, sample sizes, migraine subtypes, assessment methods, and key quantitative outcomes.

The “Healthy Vascular System” Paradox and Risk Factor Distribution. Large-scale population studies [18,19,24] present a provocative “healthy vascular system” hypothesis, where active and incident migraine are inversely associated with traditional CVRFs such as smoking, diabetes, and high SCORE2/FRS scores, as seen in Table 1. For instance, the Lifelines cohort demonstrated a stark inverse dose–response, with high-risk individuals (SCORE2 ≥ 10%) having significantly lower odds of incident migraine (OR 0.42) [19]. Conversely, Tekgol Uzuner et al. [30] and Gardener et al. [21] highlight that this relationship shifts with chronicity and age. Chronic migraine is associated with a significantly higher prevalence of HTN and diabetes across all age groups, while older Hispanic adults show a twofold increase in MA linked to long-duration HTN.

Gender-Specific Profiles and Longitudinal Cardiovascular Risks. Longitudinal data from the UK Biobank [23] and Women’s Health Study [26] suggest that migraine, particularly the MA subtype, may be associated with enhanced ASCVD risk, with hazard ratios (HRs) reaching 1.12 for total ASCVD and 3.36 per 1000 person-years for major cardiovascular disease (CVD) (refer to Table 1). Gender-specific analyses reveal that men with migraine show inverse associations with HTN and MetS, whereas women exhibit positive associations with dyslipidemia [22]. This suggests that while the “baseline” migraineur may appear vascularly healthy, the presence of the aura subtype or the progression to a chronic state may be associated with accelerated accumulation of major CV events over time [31].

Treatment Safety and Clinical Risk Management. The assessment of acute treatments [29] and hormonal therapies [25] provides critical safety insights as triptan initiation was not associated with increased 90-day CV events (aHR 0.96), even in patients with existing risk factors. Similarly, combined hormonal contraceptives (CHC) did not significantly elevate vascular risk in younger women with migraine, although MA itself remained a stronger independent risk factor for IS (HR 2.45) in non-users. Lipton et al. [27] emphasized that as CVRFs naturally increase with age, with nearly 70% of migraineurs having at least one risk factor, the risk–benefit ratio for triptans and ergots requires careful monitoring, despite the lack of acute vascular harm demonstrated in specific cohort analyses like Cloet’s [20], which found no association between MA and small vessel disease.

### 3.2. Stroke Risk and Outcomes

Table 2 summarizes the evidence on stroke risk and outcomes across 13 studies [32,33,34,35,36,37,38,39,40,41,42,43,44], detailing migraine subtype-specific associations, assessment methodologies, and principal findings.

Risk Magnitude and the Dominance of the Aura Subtype. Consistent evidence across the ARIC [32,33], NHS II [37], and large retrospective cohorts [35,38], summarized in Table 2, identifies MA as the primary driver of IS risk. Androulakis et al. reported an HR of 1.7 for MA compared to no headache [32], a risk that nearly doubles (HR 2.17) in late-onset cases [33]. This is corroborated by Kuybu et al.’s finding of a 3.23 OR for stroke in young MA patients [38]. Interestingly, the twin study by Lantz et al. [39] suggests that while migraine is a risk factor, the association may be modified by shared genetic or environmental factors, as the risk was less pronounced in within-pair analyses than in the general population.

Mechanistic Divergence: Cardioembolic vs. Atherosclerotic Pathways. The data from Table 2 suggests that migraine-related stroke is likely non-atherosclerotic. Gollion et al. [36] found a negative association between migraine and LAA (OR 0.44), suggesting different triggers. Instead, a cardioembolic mechanism is supported by Androulakis et al. (HR 3.7 for cardioembolic stroke in MA) [32] and De Giuli et al. [34], who found a powerful association between MA and PFO, particularly in women (OR 8.23). This aspect is reiterated in West et al.’s [44] study, which mentions a 79% prevalence of PFO in migraine patients. This “paradoxical embolism” pathway provides a plausible physiological bridge for the increased stroke incidence observed in MA populations, distinguishing it from traditional thrombotic stroke profiles.

The associations described in this section represent epidemiological correlations identified through a scoping review methodology. The cardioembolic and non-atherosclerotic patterns observed are consistent with, but do not establish, causal mechanisms. The language in this section has been revised to reflect descriptive evidence mapping rather than causal inference, in accordance with scoping review standards.

Recurrence, and Long-Term Outcomes. Velickovic et al. [43] suggest that acute severity, specifically MA, may be associated with short-term recurrence, showing a significant HR of 2.13 for 30-day transient ischemic attack (TIA) readmission. On the other hand, McCain et al. [41] focused on long-term outcomes, utilizing the MARS+ score to quantify lifetime risk, as they found that a score of ≥5 correlates with a fourfold increase in IS risk (HR 4.09). Together, these data suggest that while aura indicates immediate vascular vulnerability, the MARS+ score provides a more robust metric for long-term cerebrovascular prognosis.

### 3.3. Structural and Functional Vascular Assessment

Table 3 outlines the structural and functional vascular assessment results from eight studies [45,46,47,48,49,50,51,52], including measures of arterial stiffness, endothelial function, and microvascular caliber.

Macrovascular Stability and Arterial Stiffness. Current evidence from Table 3 suggests that migraine is not independently associated with accelerated macrovascular aging or subclinical atherosclerosis. Data from the ELSA-Brasil study [45] found no significant difference in carotid–femoral PWV between migraineurs and controls (8.01–8.11 m/s vs. 8.67 m/s, *p* > 0.05), indicating preserved arterial compliance. Furthermore, multiple studies [46,47,50] consistently suggest that migraineurs do not exhibit higher coronary or intracranial calcification. Wen et al. [52] even observed a negative association, where migraineurs had significantly less intracranial internal CAC (log-transformed difference −0.19; 95% CI −0.29 to −0.08) compared to non-migraineurs. These findings are consistent with the hypothesis that the increased stroke risk in migraine may involve mechanisms other than large-vessel structural atherosclerosis.

Endothelial Dysfunction and Microvascular Caliber. While migraine, particularly with aura, is often linked to vascular impairment, population-based functional and structural assessments yield largely null results regarding systemic microvascular changes. In the HUNT3 Study [48], as seen in Table 3, FMD, a gold standard for endothelial function, showed no significant impairment in migraineurs (MA: 5.08% vs. MwA: 5.31%). Similarly, the Rotterdam Study [51] found no association between migraine and retinal microvascular caliber or retinopathy (OR 1.09; 95% CI 0.62–1.92). These data suggest that if endothelial dysfunction exists, it may be localized to the cerebral circulation or transiently expressed during attacks rather than manifesting as a persistent systemic microvascular trait.

Cerebral Autoregulation and Vasoreactivity. Despite the lack of structural macrovascular damage, migraineurs exhibit distinct clinical signs of autonomic and peripheral vasoreactivity imbalances. Linstra et al. [49] identified a significant prevalence of “cold extremities” among migraineurs (OR 2.3; 95% CI 1.4–3.7), a marker of peripheral vascular dysregulation that correlates with higher attack frequency. When combined with the findings from van Os et al. [50], which showed lower rates of intracranial atherosclerosis in migraineurs (62% vs. 79% in controls), the evidence points toward a functional, rather than structural, hemodynamic impairment. This suggests that the migraine brain operates within a framework of altered vasoreactivity and impaired hemodynamic control, which may predispose individuals to ischemia despite having “healthier” looking vessels.

### 3.4. Inflammatory and Circulating Biomarkers

Table 4 presents the inflammatory and circulating biomarker data from two studies [12,53], focusing on markers such as hs-CRP and fibrinogen in migraineurs compared to controls.

Chronic Systemic Proinflammatory State. The research by Avci et al. [12] and Tietjen et al. [53], both summarized in Table 4, provides strong evidence for a persistent proinflammatory environment in migraineurs, even outside of acute attacks. Avci et al. [12] reported hs-CRP levels more than double those of controls (1.94 vs. 0.82 mg/L), while Tietjen et al. [53] (CAMERA study) found significantly elevated hs-CRP (3.43 mg/L) particularly in women with MA. These findings suggest that migraine is not merely an episodic pain disorder, but a condition characterized by low-grade, chronic systemic inflammation.

Interictal Hypercoagulability. Tietjen et al.’s analysis of the CAMERA cohort [53] further reveals a state of hypercoagulability that persists interictally (>3 days from an attack). Migraineurs showed significantly higher levels of fibrinogen (316 vs. 298 mg/dL) and Factor II activity compared to controls. This chronic elevation of hemostatic markers, coupled with the inflammatory markers, creates a “pro-thrombotic” milieu that may contribute to the long-term vascular risks identified in Categories 1 and 2 (Table 1 and Table 2).

Clinical Implications of Biomarker Elevation. The consistency between these two studies [12,53], despite their different populations, highlights that circulating biomarkers like hs-CRP and Fibrinogen are objective indicators of the vascular stress associated with migraine. The fact that these markers remain elevated between attacks suggests that the “vascular burden” of migraine is continuous, providing a biological rationale for why migraine (especially with aura) functions as an independent CVRF.

### 3.5. Radiological and Neuroimaging Findings

Table 5 summarizes the radiological and neuroimaging findings from two studies [12,54], highlighting the prevalence and characteristics of WMH in migraine patients.

The neuroimaging analysis presented in this section covers exclusively the presence and burden of WMH as identified on brain MRI in adult migraine patients. This section does NOT encompass other morphological and functional brain abnormalities that may be documented in migraine patients, such as cortical thinning, hippocampal volume changes, functional connectivity alterations, perfusion abnormalities, silent infarcts beyond WMH, or other structural lesions. The scope was strictly limited to WMH as the primary neuroimaging outcome consistent with our predefined search strategy and eligibility criteria.

Prevalence and Burden of White Matter Hyperintensities. Neuroimaging findings indicate a significantly higher burden of subclinical brain lesions in migraineurs; the data is presented in Table 5. Avci et al. [12] found that 31.9% of migraine patients had WMHs compared to only 9.7% of controls (OR 4.35), with lesions predominantly located in the frontal lobe. Vijiaratnam et al. [54] reported even higher prevalence rates (up to 41% in MWoA and 39% in MA), though notably found no significant difference between migraine subtypes, suggesting that WMH accumulation may be a general feature of the migraine brain.

Correlation with Traditional Risk Factors vs. Migraine Specifics. A critical divergence appears regarding the etiology of these lesions. While Avci et al. [12] found no correlation between inflammatory markers (hs-CRP) and WMH burden, Vijiaratnam et al. [54] observed that WMHs were more strongly associated with advancing age and traditional CVRFs than with migraine characteristics themselves. This suggests that while migraineurs have more lesions, the progression of these lesions might still be governed by standard vascular aging processes, rather than exclusively by the inflammatory or “migraine-specific” mechanisms suggested in Category 4.

Diagnostic and Clinical Utility of Routine Imaging. Both studies, particularly Vijiaratnam et al. [54], conclude that routine neuroimaging (CT or MRI) for migraine diagnosis or subtype differentiation has limited clinical value. In Vijiaratnam et al. [54]’s cohort, carotid Doppler and brain imaging yielded almost no clinically significant findings that altered management. Therefore, while WMHs are a frequent and objective finding on MRI, they should be viewed as markers of “cumulative vascular exposure” rather than acute diagnostic tools.

### 3.6. Genetic and Molecular Biomarkers

Table 6 presents the genetic and molecular biomarker findings from three studies [55,56,57], examining polymorphisms in the NO pathway and vascular wall integrity genes.

The genetic analysis presented in this section covers exclusively single nucleotide polymorphisms (SNPs) in the neuronal nitric oxide synthase gene (NOS1, specifically rs693534 and rs7977109) and the endothelial nitric oxide synthase gene (NOS3, specifically rs2070744), as well as the LRP1 rs11172113 variant. This section does NOT encompass other genetic factors that may be implicated in migraine pathogenesis or cardiovascular comorbidities. The scope was strictly limited to NO pathway and vascular wall integrity genes identified through our predefined search strategy.

The Nitric Oxide Pathway: NOS3 and NOS1 Variations. Genetic investigations into the NO pathway, a key regulator of vascular tone, have largely yielded negative results regarding primary disease susceptibility. García-Martín et al. [55,56] analyzed functional polymorphisms in both endothelial (NOS3) and neuronal (NOS1) NOS genes. Neither study (Table 6) found a significant association between these single nucleotide polymorphisms (SNPs) and overall migraine risk. However, the NOS3 CC genotype was more frequent in those with a family history of migraine, suggesting that NO-related genes might play a role in the hereditary clustering of the disorder rather than being universal risk alleles.

Vascular Wall Integrity and Syncope Risk (LRP1). Kuan et al. [57] provides a specific genetic link between vascular health and comorbid symptoms. The study identified that the rs11172113 polymorphism in the LRP1 gene, crucial for vascular wall integrity, was significantly associated with syncope risk in migraineurs (OR 4.00). This allele is linked to lower gene expression in vascular tissues, suggesting that a genetically determined “vascular weakness” may predispose certain migraineurs to autonomic and syncopal complications.

Synthesis of Genetic Findings. Overall, Category 6 (Table 6) indicates that while common variants in the NO pathway are not primary drivers of migraine, specific loci like LRP1 highlight the importance of “vascular wall health” in the migraine phenotype. This reinforces the concept that the vascular risk in migraine may be rooted in structural and functional genetic predispositions that affect the entire circulatory system, rather than just the trigeminovascular system.

### 3.7. Neurocognitive Profiles

Table 7 summarizes the neurocognitive profile data from two studies [13,58], describing cognitive performance differences associated with migraine subtypes.

Absence of Long-Term Dementia Risk. The ARIC-NCS study [13] provides robust, long-term reassurance regarding cognitive decline. Over a 21-year follow-up of 12,495 participants, no association was found between a history of migraine and the incidence of dementia (HR 1.04). Even when stratifying for the aura subtype (HR 1.12), the results remained non-significant, suggesting that the structural brain changes observed in Category 5 (WMHs) do not inevitably lead to clinical cognitive failure.

The “Migraine-Cognition Paradox”: Superior Performance. Paradoxically, the Rotterdam Study [58] found that migraineurs performed better on cognitive tests than non-migraineurs. Migraineurs, especially those with aura, showed higher scores in MMSE (0.39 points higher) and global cognition. This was consistent across both sexes, suggesting that the migraine brain may possess unique neuroplastic or compensatory mechanisms, or that the “healthier” baseline profile of episodic migraineurs (Category 1—Table 1) translates into better cognitive preservation.

Conclusion on Cognitive Outcomes. Synthesizing Categories 5 and 7 (Table 5 and Table 7) reveals a fascinating dichotomy: while the migraine brain is more likely to harbor structural lesions (WMHs and silent infarcts), it is remarkably resilient to cognitive decline. The “migraine-cognition paradox” suggests that the vascular and radiological risks associated with migraine do not translate into the neurodegenerative outcomes typical of traditional vascular disease, marking migraine as a unique and complex vascular phenotype.

### 3.8. The Multiverse of Migraine: Intersecting Domains of Vascular Risk and Paradoxical Resilience

**Figure 2:**
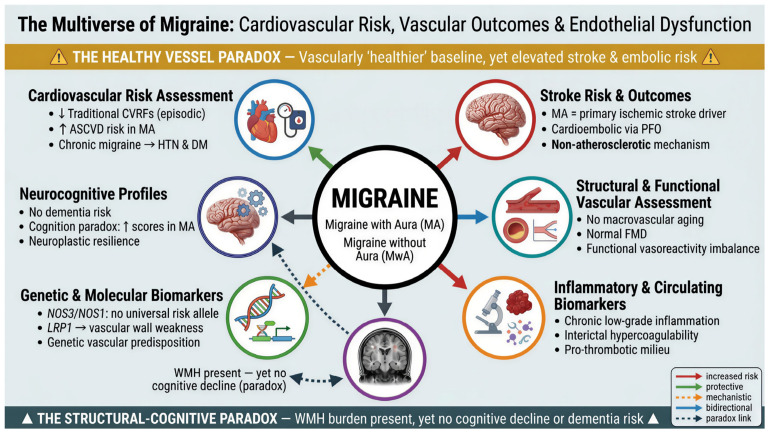
The Multiverse of migraine: intersecting domains of vascular risk and paradoxical resilience (illustration created by the authors using Microsoft PowerPoint). Abbreviations: CVRFs—cardiovascular risk factors; ASCVD—atherosclerotic cardiovascular disease; HTN—hypertension; DM—diabetes mellitus; PFO—Patent Foramen Ovale; FMD—flow-mediated dilation; WMH—white matter hyperintensities; NOS3—Nitric Oxide Synthase 3 (endothelial NOS gene); NOS1—Nitric Oxide Synthase 1 (neuronal NOS gene); LRP1—Low-Density Lipoprotein Receptor-Related Protein 1.

Figure 2 illustrates the intersecting domains of vascular risk and paradoxical resilience in migraine, depicting the two central paradoxes that define the migraine vascular phenotype.

This conceptual framework synthesizes seven interconnected domains of migraine-related vascular pathophysiology identified in this scoping review: (1) CV risk assessment, (2) stroke risk and outcomes, (3) structural and functional vascular assessment, (4) inflammatory and circulating biomarkers, (5) radiological and neuroimaging findings, (6) genetic and molecular biomarkers, and (7) neurocognitive profiles.

The diagram (Figure 2) illustrates two central paradoxes that define the migraine vascular phenotype. The Healthy Vessel Paradox reflects the inverse association between episodic migraine and traditional CVRFs, despite elevated stroke and cardioembolic risk, particularly in MA. The Structural-Cognitive Paradox highlights the dissociation between increased WMH burden and preserved, or even superior, cognitive performance, with no long-term dementia risk. Together, these domains and paradoxes underscore migraine as a unique vascular condition characterized by functional dysregulation and paradoxical embolism, rather than classical atherosclerotic disease, challenging conventional CV risk stratification models.

## 4. Discussion

The findings of this scoping review should be interpreted within the broader framework of migraine as a comorbid condition with systemic vascular implications. The bidirectional relationship between migraine and CVD, wherein migraine both reflects and contributes to underlying vascular dysfunction, positions this disorder at the intersection of neurology and internal medicine, with direct relevance to clinical governance and patient management across specialties.

This scoping review synthesized data from 43 studies to explore the association between migraine and CV risk across seven interconnected thematic areas. The following discussion addresses each domain in turn, drawing on evidence from primary studies and existing reviews to contextualize the findings within the broader landscape of migraine-related vascular pathology.

### 4.1. Migraine and Cardiovascular Risk

While many traditional CVRFs and migraine have been studied, there have been heterogeneous results and differences between genders. There are differences between MA and MO (for example, MA is associated with a higher vascular risk), as well as gender. Several CV factors are discussed in this paper, including age, HTN, ASCVD, smoking, and diabetes. The risks of treatment like triptans and hormonal contraceptives are also discussed along with the necessity of monitoring their use. Considering the increase in CV risk with age, care must be taken with patients who have at least one risk factor.

The present review highlights a paradoxical inverse association between active migraine and traditional CVRFs in general populations: active migraineurs exhibit lower odds of high SCORE2 (OR 0.43) and FRS (OR 0.64), with reduced smoking and diabetes prevalence [18,24]. Yet, this “healthy baseline” profile does not confer vascular protection, as Schürks et al. [3] stated that MA independently doubles IS risk (RR 2.16, 95% CI 1.53–3.03), even absent classical risk factor burden. This dissociation implies that MA operates through aura-mediated pathways like CSD or/and endothelial dysfunction and it can be undetected by conventional scoring tools, a vulnerability that becomes clinically apparent with aura emergence or progression to chronic migraine [26,30].

Traditional CVRFs, rather than absent, act as potent amplifiers of an already elevated baseline risk in MA patients. Schürks et al. [3] showed that smoking raises IS risk nearly ninefold in migraineurs (RR 9.03), while oral contraceptive use produces a sevenfold increase (RR 7.02), peaking in women under 45 (RR 3.65). This aligns with the gender-specific patterns identified here: men show inverse associations with HTN and MetS [22], whereas women with MA accumulate major CV events [26], with long-term ASCVD risk independently elevated [23]. These findings collectively indicate that the paradox reflects not true vascular health, but a risk architecture in which aura-mediated and chronicity-driven mechanisms remain systematically underestimated by classical CV scoring tools.

Despite the breadth of evidence synthesized, the included studies present notable methodological gaps including inconsistent migraine subtype classification, underrepresentation of male and non-Western populations, and the absence of prospective data tracking the episodic-to-chronic migraine transition. There is a further need for longitudinal, sex-stratified cohort studies that integrate aura status and migraine chronicity as independent variables within validated CV risk prediction models.

### 4.2. Migraine and Stroke

Cohort and meta-analytic evidence consistently suggest an association between MA and increased IS risk. Effect sizes show remarkable alignment: ARIC HR of 1.67 [32] and 2.17 for late-onset MA [33] converge with pooled estimates of RR 2.16 [3] and OR 2.32 [4]. Risk is amplified in younger populations, with OR 3.23 in MA patients aged 18–44 years [38], corroborated by meta-analytic OR 2.39 in adults ≤ 45 years [4]. MO shows no significant independent stroke risk (pooled RR 1.23) [3], though an emerging OR of 1.77 [4] warrants continued surveillance.

The mechanistic profile diverges sharply from atherosclerotic pathways. Androulakis et al. [32] documented a cardioembolic HR of 3.7, while Gollion et al. [36] reported a negative LAA (OR 0.44), supporting non-atherosclerotic, cardioembolic mechanisms. PFO emerges as a key structural mediator, as De Giuli et al. [34] reported OR 8.23 in women, and West et al. identified 79% PFO prevalence in migraineurs [44], alongside CSD and endothelial dysfunction as pathophysiological links [5]. However, Davis et al. [7] introduce an important caveat as PFO–stroke associations attenuate substantially in population-based designs, suggesting that hospital-based estimates may reflect selection bias. Notably, late-onset MA (≥50 years) confers the highest long-term risk (HR 2.17) [33], with this subgroup carrying disproportionate burden.

Beyond stroke, migraine functions as a broader systemic vascular disorder [37], though endpoint-specific associations vary as angina risk was confirmed but MI risk was non-significant [3]. Long-term cerebrovascular risk is stratifiable: HR 2.13 for 30-day TIA readmission [43], fourfold increase in IS for MARS+ ≥ 5 (HR 4.09) [41], and absolute IS incidence reaching 47.2 per 1000 person-years in high-risk MA subgroups [35]. Atrial fibrillation (AF) represents an additional parallel cardioembolic pathway, with HR of 1.30 for incident AF in MA [42]. Notably, Li et al. [40] demonstrate the strongest stroke association in adults ≥ 65 years (OR 1.81) with few vascular risk factors, consistent with an independent vascular pathway persisting across the lifespan. Finally, Dzator et al. [6] provide physiological corroboration through elevated arterial stiffness and impaired cerebrovascular responsiveness in the posterior circulation, paradoxically more pronounced in MO, suggesting subclinical vascular dysfunction across both subtypes.

Critical gaps persist regarding the temporal dynamics of migraine–stroke risk and the interplay between genetic and acquired contributors. Lantz et al. [39] suggest that shared familial factors partially confound this association, though migraine retains independent predictive value at the population level. The inconsistency between case–control and population-based PFO–stroke estimates [7] further underscores the need for rigorous prospective studies to establish causal pathways and quantify attributable risk.

### 4.3. Migraine and Endothelial Dysfunction

The convergence of null macrovascular findings across primary studies, including preserved PWV [45], absence of excess coronary calcification [46,47], reduced ICAC [50,52], and normal FMD [48], contrasts sharply with the consistent elevation of endothelial activation biomarkers documented in systematic reviews [8,9]. This divergence suggests that migraine-associated vascular risk operates through transient functional impairment rather than fixed structural atherosclerosis. Importantly, a meta-analysis by Pang et al. [59], included here as contextual evidence rather than as a primary study within the scoping review corpus, demonstrated significant retinal microvascular rarefaction in migraineurs (superficial macular vessel density SMD = −0.30; foveal avascular zone area SMD = +0.56), indicating that microvascular, not macrovascular, pathology may be the primary substrate. From an internal medicine perspective, this functional microvascular impairment, persistent even interictally, reinforces the need for systematic CV screening in migraine patients, particularly in ageing populations where comorbidity burden accumulates. Van Os et al. [50] found no excess LAA in migraineurs, while Wen et al. [52] reported a significant inverse association ICAC (β −0.19; 95% CI −0.29 to −0.08). The clinical marker of cold extremities (OR 2.3; 95% CI 1.4–3.7) [49] further corroborates a functional autonomic and peripheral vasoreactivity imbalance correlating with attack frequency, reinforcing the framework of altered cerebral autoregulation without structural macrovascular damage.

The endothelial dysfunction patterns described here are based on cross-sectional population studies and should be interpreted as descriptive associations rather than established pathophysiological mechanisms. No included study directly demonstrated causal endothelial injury attributable to migraine. These findings map the current evidence landscape and identify research gaps rather than confirming causal links.

Mechanistically, these null findings align with the inherent arteriopathy hypothesis [11], wherein smaller-diameter muscular arteries and elevated serum elastase paradoxically confer arterial distensibility rather than stiffness. Apelbaum et al. [45] proposed that elevated NO during headache-free periods induces cerebral vasodilation, explaining preserved PWV despite episodic vascular dysfunction. Cuciureanu et al. [10] further emphasized that CSD-triggered CGRP release may drive transient vasodilation and inflammation without chronic structural remodeling. Reduced EPC counts [8] may represent precursors of later dysfunction, yet cross-sectional data from Larsen et al. [48] and Wen et al. [51] show no systemic impairment in retinal caliber or brachial FMD, suggesting that endothelial dysfunction, if present, is either localized to the cerebral circulation or expressed exclusively during attacks.

As critical gaps we acknowledge that the eight primary studies are cross-sectional and cannot establish temporality and no study directly measured cerebral endothelial function or attack-phase vascular parameters.

### 4.4. Migraine and Biomarkers

Only two studies met the inclusion criteria for the association between circulating biomarkers, such as hs-CRP, and migraine, suggesting that this is a relatively new, yet still underexplored, area of interest. Notably, our biomarker selection was strictly dictated by a vascular-centric search strategy focused on endothelial dysfunction. Consequently, classic thrombophilia profiles fell outside our predefined search blocks—consistent with key included studies (Avci et al. [12]; Tietjen et al. [53]), both of which framed their biomarker panels within endothelial activation and vascular stress rather than inherited thrombophilic disease.

The results of the study by Tietjen et al. [53], which evaluated biomarkers from participants in the Dutch CAMERA 1 study, show that selected biomarkers of vascular disease are elevated in migraine, especially in subgroups most strongly associated with IS risk—those with aura and women. The authors demonstrated that, within the MA subgroup, aura, attack frequency, and disease duration since onset, rather than headache, were associated with elevated inflammatory biomarkers, including fibrinogen, hs-CRP, vWF Ag, and D-dimer. The total number of years with aura attacks was a significant predictor of hs-CRP (β = 0.32, *p* < 0.00). Elevated fibrinogen and factor II levels were strongly associated with migraine, and this association remained robust even after controlling traditional stroke risk factors. In addition, the mean number of aura attacks was a significant predictor of hs-CRP (β = 0.39, *p* < 0.001), vWF Ag (β = 0.22, *p* = 0.007), D-dimer (β = 0.26, *p* = 0.001), and fibrinogen (β = 0.16, *p* = 0.036).

In the analysis of the subgroup of young adult women (19–34 years), those with MO had borderline higher hs-CRP levels than non-migraineurs and those with MA (1.01 mg/L vs. 0.81 and 0.75 mg/L, *p* = 0.08 and *p* = 0.08). Also, in the study of Avici et al., serum hs-CRP levels were significantly higher in migraine patients than in control subjects. The association of hs-CRP with migraine had been previously demonstrated in other studies, including in that with MO and with MA. The study by Avci et al. didn’t find a statistically significant correlation between hs-CRP levels and WMHs in migraine and control subjects (r = 0.155; *p* > 0.001). Also, the study suggest that investigating additional markers of endothelial dysfunction or inflammation, such as tissue plasminogen activator antigen, vWF activity, homocysteine levels, or inflammatory cytokine concentrations, may be useful for better evaluating the role of hs-CRP in the development of WMHs in patients with migraine. The absence of an association between hs-CRP levels and WMHs suggests that hs-CRP does not influence the brain pattern or severity of these lesions in patients with migraine, which may instead be related to the disease itself and possibly genetically determined [12]. In the Tietjen et al. study [53], plasma levels of vWF antigen, a well-established marker of endothelial activation, correlated positively with all other biomarkers, as well as with aura frequency and duration since onset, in the MA cohort. vWF antigen levels were, however, not elevated in migraine compared with controls. There was no correlation between hs-CRP and headache characteristics (r < 0.042; not significant).

Inflammatory and circulating biomarkers, particularly hs-CRP and fibrinogen, are consistently elevated in migraineurs compared to controls, even interictally, with stronger associations observed in the MA subgroup. The prothrombotic and proinflammatory state documented here provides a plausible biological substrate for the excess CV risk identified in Categories 1 and 2. However, the limited number of studies meeting inclusion criteria (*n* = 2) and the absence of longitudinal biomarker data preclude definitive conclusions regarding the temporal dynamics of biomarker elevation relative to attack cycles.

### 4.5. Migraine and Neuroimaging

Paraclinical investigations, including brain MRI, brain CT, and carotid Doppler ultrasonography, were performed more frequently in patients with MA than in those MO. The more frequent use of imaging investigations in patients with MA may be explained by the presence of focal neurological manifestations associated with aura, which often prompt a clinical approach like that used in suspected strokes. Among the cerebral abnormalities identified on MRI in patients with migraine, WMHs were the most common findings [54]. In the study conducted by Avci et al. [12], WMHs were predominantly identified in the supratentorial region (97%), whereas only a small proportion of patients had infratentorial lesions (3%). Regarding lesion size, most WMHs were ≤3 mm in diameter (91%), whereas a smaller proportion measured between 4 and 9 mm (9%). The presence and number of WMHs per subject, including juxtacortical, subcortical, and periventricular lesions, were significantly higher in patients with migraine than in control subjects (*p* ≤ 0.001). In both the migraine and control groups, WMHs were most frequently located in the frontal lobe. Unlike the control group, in which WMHs were detected only in the subcortical region, migraine patients also showed juxtacortical and periventricular lesions. The frequencies of juxtacortical and subcortical WMHs were similar between patients with MA and those without aura, whereas periventricular WMHs were observed exclusively in patients with MA. Prevalence of WMHs was 4.35 times higher in patients with migraine than in control subjects. These findings, obtained in a cohort of patients without known vascular risk factors or inflammatory diseases, support the robustness of the reported observations. However, no statistically significant correlation was found between hs-CRP levels and WMHs in either group (r = 0.155, *p* > 0.001) [12].

We emphasize that our neuroimaging review focused strictly on WMH as the primary outcome, and did not encompass other morphological alterations, perfusion abnormalities, or functional connectivity changes.

WMHs represent a consistent neuroimaging finding in migraineurs, present in approximately one-third to two-fifths of patients across both subtypes. However, the progression of WMH burden appears to be governed more by age and traditional CVRFs than by migraine-specific mechanisms, as evidenced by the absence of subtype-specific differences and the lack of correlation with interictal inflammatory markers. This finding has direct clinical implications such as that routine neuroimaging for migraine subtype differentiation carries limited diagnostic yield, and WMHs should be interpreted as markers of cumulative vascular exposure rather than as independent drivers of neurodegeneration.

### 4.6. Migraine and Genetics

NO has several important biological functions, including the inhibition of platelet aggregation and the maintenance of arterial vasodilatory tone. The possible association between functional SNPs in the neuronal NOS gene and migraine risk has been investigated repeatedly by García-Martín et al. [55,56]. In their first case–control study (2015) [55], the authors investigated the frequencies of the NOS1 rs7977109 and rs693534 genotypes and allelic variants, which are related to the neuronal isoform of NOS. In their second study (2019) [56], they examined the frequency of the NOS3 rs2070744 genotype, which is associated with the endothelial isoform, namely, eNOS.

In the 2015 study [55], genotype frequencies were not influenced by sex, age at migraine onset, positive family history of migraine, or the presence or absence of aura in either group. In the conducted study by García-Martín et al. [56], the frequencies of rs2070744 genotypes and allelic variants in the NOS3 gene, which encodes the eNOS protein, were similar in patients with migraine and controls, with no differences between MA and MO, and no influence of sex or age at migraine onset. A higher frequency of the NOS3 rs1799983 TT genotype was observed in patients with headache duration exceeding 24 h compared with those with shorter migraine attacks, suggesting a possible role of the NOS3 gene in migraine. In addition, an association was observed between the rs2070744CC genotype and the rs2070744C allele, and with a positive family history of migraine, although the statistical significance of this finding was not strong enough. Nevertheless, in the Caucasian population studied, no association was found between NOS3 rs2070744 variants and the risk of developing migraine [56]. Another study investigating vascular risk factors associated with migraine was conducted by Kuan et al. [57], who reported that patients with MA had an approximately 1.8-fold higher risk of syncope compared with those without aura, a finding the authors suggested might be explained by endothelial dysfunction associated with MA. In addition, the exploratory genetic analysis identified a recessive genotypic association between rs11172113 in the LRP1 gene and syncope risk (OR = 4.00; 95% CI: 1.03–15.45; *p* = 0.045) among individuals carrying the C risk allele. By contrast, no evidence of an association with syncope risk was found for the other susceptibility loci analyzed, namely, rs10166942 in TRPM8, rs655484 in DLG2, and rs3781545 in GFRA1 [57].

It should be noted that this review addressed solely the link between NOS variants and migraine susceptibility, rather than broader genetic factors.

Common polymorphisms in the NO pathway (NOS1 rs7977109, NOS3 rs2070744) are not primary drivers of migraine susceptibility at the population level. The borderline association between the NOS3 CC genotype and positive family history, and the significant LRP1 rs11172113–syncope association (OR 4.00), suggest that genetic vascular risk in migraine may operate through structural predispositions affecting vascular wall integrity rather than through NO-mediated vasomotor dysregulation alone. The small sample sizes of the included studies limit statistical power for detecting modest genetic effects, and replication in larger, multiethnic cohorts is warranted.

### 4.7. Migraine and Cognitive Profiles

A large study with a 21-year follow-up conducted by George et al. [13] reported a cumulative dementia incidence of 16.7% (233/1397) among participants with migraine. In the ARIC study, however, no statistically significant difference was observed in the overall incidence of dementia between individuals with migraine symptoms (16.7%), those with severe non-migraine headache (15.8%), and those without headache (18.5%). This lack of association was evident in both men and women. Although migraine has been linked to cerebrovascular lesions, migraine-related brain changes appear to remain stable over time and may, therefore, not contribute substantially to the pathophysiology of dementia later in life [13]. Furthermore, the study by Wen et al. [58], conducted in 6708 participants with definite or probable migraine and including middle-aged and older adults, showed that individuals with migraine, particularly those with MA, achieved higher MMSE and g-factor scores than non-migraineurs. Although some previous studies have reported poorer performance in migraine patients, especially in processing speed, these discrepant findings may be explained by differences in study populations, clinical versus population-based recruitment, participants’ age, and the methods used to assess migraine. In addition, variability across studies may reflect the limitations of the MMSE, a relatively crude global cognitive screening tool with limited sensitivity for detecting subtle cognitive differences [58].

Despite a significantly higher burden of structural brain lesions, migraineurs do not demonstrate increased dementia risk and may, paradoxically, perform better on global cognitive assessments than non-migraineurs. This dissociation between structural and functional brain outcomes suggests that the migraine brain may engage neuroplastic or compensatory mechanisms that buffer against neurodegeneration, or, alternatively, that the “healthy vascular baseline” of episodic migraineurs translates into preserved cognitive reserve. The MMSE-based assessment used in Wen et al. [58] may, however, lack sensitivity for detecting subtle domain-specific deficits, and future studies should incorporate comprehensive neuropsychological batteries.

### 4.8. Chronic Migraine—A Special Consideration

Across the 43 included studies and 7 thematic domains, evidence specifically addressing chronic migraine (CM) as a distinct clinical entity remains notably sparse.

In the cardiovascular risk domain (Table 1), only a limited number of studies explicitly stratified outcomes by CM status: Tekgol Uzuner et al. [30] reported that CM patients had higher prevalence of CVRFs compared to episodic migraine, while data from the UK Biobank [23] and the Women’s Health Study [26] primarily captured episodic migraine populations, with CM subgroups underrepresented.

In the stroke domain (Table 2), none of the 13 included studies were designed primarily for CM populations; the available data address MA and broader migraine risk categories [35,38], leaving the CM-specific stroke burden unquantified.

In the neuroimaging domain (Table 5), Avci et al. [12] documented a significantly higher WMH prevalence in migraine patients compared to controls, though no CM-specific neuroimaging data were reported. In the biomarker domain (Table 4), Tietjen et al. [53] demonstrated that within the MA subgroup, attack frequency and disease duration were associated with elevated hs-CRP and fibrinogen, though CM was not explicitly studied as a separate entity. The genetic and cognitive domains (Table 6 and Table 7) did not include CM-specific analyses.

This pattern of evidence reveals a critical gap: CM, defined as ≥15 headache days/month for >3 months with migraine features on ≥8 days/month (ICHD-3) [17] carries a substantially higher disability burden, greater analgesic overuse risk, and more pronounced psychiatric and vascular comorbidities than episodic forms, yet remains systematically underrepresented in large-scale vascular research. From a clinical standpoint, neurologists and vascular specialists managing CM patients should apply heightened vigilance regarding cardiovascular risk assessment, periodic biomarker monitoring (hs-CRP, fibrinogen), and neuroimaging surveillance for WMH progression. Future research must prioritize dedicated CM cohorts with rigorous ICHD-3 classification, longitudinal cardiovascular outcome adjudication, and systematic vascular biomarker profiling to generate the CM-specific evidence base currently absent from the literature.

### 4.9. Integrative Synthesis and Clinical Implications

Taken together, the seven thematic domains examined in this scoping review converge on a coherent, if paradoxical, vascular phenotype. Episodic migraine, particularly in its active form, presents with a “healthy vessel” profile at the macrovascular level represented by preserved arterial compliance, absent coronary calcification, and inverse associations with traditional CVRFs. Yet this apparent vascular health coexists with a persistent proinflammatory and prothrombotic interictal state, functional microvascular rarefaction, a substantially elevated WMH burden, and, critically, an independent, non-atherosclerotic IS risk mediated by cardioembolic mechanisms including PFO and AF. The structural-cognitive paradox further distinguishes migraine from classical vascular disease, where WMHs accumulate without conferring dementia risk, suggesting that the migraine brain retains functional resilience despite structural vulnerability.

These findings carry concrete clinical implications for multidisciplinary practice:

(1) Cardiovascular risk scoring: Standard tools (SCORE2, FRS) systematically underestimate vascular risk in active migraineurs, particularly those with aura. Clinicians should apply aura status as an independent risk modifier in CV risk stratification, consistent with current ESC guidelines.
(2) PFO screening: Given the high PFO prevalence in migraineurs with cryptogenic stroke (79% [44]) and the strong MA–PFO interaction (OR 8.23 in women [34]), systematic PFO evaluation should be considered in young patients with MA presenting with cryptogenic ischemic events.
(3) Biomarker monitoring: Interictal elevation of hs-CRP and fibrinogen in migraineurs, particularly women with MA, suggests a role for periodic biomarker surveillance in high-risk subgroups, even in the absence of acute CV events.
(4) Hormonal and acute therapies: Combined oral contraceptives should be used with caution in women under 45 with MA, given the amplified stroke risk. Triptan use, however, was not associated with increased short-term CV events [29] and should not be withheld solely on CV grounds in the absence of established contraindications.

### 4.10. Limitations

The present scoping review has several limitations that should be acknowledged. From a methodological standpoint, the quality of the included evidence varies considerably: the majority of studies are cross-sectional or retrospective in design, precluding causal inference; migraine subtype classification was inconsistent across studies, with only approximately 80% distinguishing between MA and MO according to ICHD criteria [17]; and several studies relied on self-reported or administrative migraine diagnoses, introducing potential misclassification bias. Hospital-based and clinic-based recruitment strategies may further inflate effect size estimates relative to population-based designs, as demonstrated by the attenuation of PFO–stroke associations in population-based, compared to case–control, studies [7]. These methodological heterogeneities should be considered when interpreting the pooled narrative findings presented in this review.

Furthermore, in strict alignment with the scoping review framework, the objective of this review is confined to mapping the landscape of existing evidence and descriptive associations across domains. This methodology does not permit the synthesis of direct causal inferences or the establishment of definitive pathophysiological mechanisms.

The search was conducted exclusively in PubMed/MEDLINE, which may have resulted in the omission of relevant studies indexed in other databases, and, also, no grey literature was included. The restriction of the literature search to PubMed/MEDLINE as the sole bibliographic database, without supplementary retrieval from additional sources (e.g., Embase, Scopus, CINAHL) or grey literature, represents a key methodological limitation. Studies indexed exclusively in other databases may have been missed, potentially underrepresenting interdisciplinary or regional literature. The use of free-text terms, rather than MeSH headings, while intentionally designed to enhance sensitivity toward emerging terminology, may introduce variability in retrieval consistency. However, consistent with the defining purpose of scoping reviews, which are to “map evidence on a topic and identify main concepts, theories, sources, and knowledge gaps” rather than to achieve exhaustive retrieval [16], the findings could be interpreted as a focused evidence map of the PubMed-indexed literature. Future work should incorporate multidatabase strategies to build upon and validate the evidence presented here.

The ≥400 participant threshold was applied as a pragmatic eligibility criterion, and the review was retrospectively registered. No formal quality assessment or risk of bias evaluation was performed on the included studies, which is consistent with scoping review methodology, but limits the ability to draw conclusions about evidence quality. Furthermore, significant clinical and methodological heterogeneity across included studies precluded cross-study comparisons. The ≥400 participant cut-off, while justified as a proxy for statistical power and cross-domain comparability, restricts the capacity of this evidence map to capture early-stage mechanistic pathways, advanced neuroimaging findings, and exploratory biomarker data typically evaluated in small-sample laboratory or proof-of-concept designs. These study types represent a complementary and scientifically valuable evidence base that falls outside the operational scope of the current review. Future targeted reviews focusing specifically on the mechanistic, imaging, and biomarker literature in this field are warranted to complement the population-level map presented here. Furthermore, the associations described throughout this review should not be interpreted as proven causal links; they represent descriptive associations identified through a structured evidence-mapping exercise, consistent with the inherent methodological scope of scoping review methodology.

Some studies did not consider the classification according to the ICHD of migraine, thereby generating a bias regarding underlying mechanisms. Studies must also consider the chronic nature of the disorder, which can accelerate the accumulation of major CV events in time.

The presented correlations should not be interpreted as proven causal links between migraine subforms and specific vascular end-points. They represent descriptive associations identified through a structured evidence-mapping exercise. In several sections, the language used to describe these associations has been revised to ensure that evidence mapping is clearly separated from causal interpretation.

Finally, the limited number of studies available in Categories 4, 5, and 7 (*n* = 2 each) constrains the conclusions that can be drawn in these domains, highlighting important gaps in the current literature that warrant further investigation.

## 5. Conclusions

This scoping review mapped evidence across 43 studies and 7 thematic domains to address the primary research question: What is the current state of evidence on the association between migraine and CV risk in adult populations? The evidence consistently positions migraine, and particularly MA, as a disorder with systemic vascular implications that extend well beyond its neurological profile.

As migraine is believed to be associated with vascular mechanisms, researchers consider several CVRFs to be potentially important. According to some studies, MA has been associated with higher CV risk in women, whereas chronic migraine sufferers have higher CVRFs of all ages. Furthermore, migraine has a gender-specific association, such as lipids in women and CVRFs (except DBP) are inversely related to migraine in females. There is an association between migraine and long-term ASCVD, and smoking can modify the migraine–stroke relationship in the elderly as well. Other authors have also pointed out that the aura subtypes accelerate the accumulation of CV events over time. MA is also an independent, non-atherosclerotic risk factor for IS via cardioembolic mechanisms, including PFO and AF, with risk highest in late-onset and younger MA patients. This vulnerability is amplified by underlying functional microvascular dysregulation and elevated endothelial biomarkers, underscoring a distinctly functional, rather than structural, vascular substrate. These findings collectively suggest that CVD may represent not merely a risk association, but a clinically relevant comorbidity of migraine, one that may benefit from integrated, multidisciplinary management strategies bridging neurology, internal medicine, and CV medicine.

Selected vascular disease biomarkers are elevated in migraine, particularly in women and in subgroups with MA. Within the MA subgroup, aura, attack frequency, and disease duration since onset are associated with increased levels of inflammatory biomarkers. Although serum hs-CRP levels were significantly higher in patients with migraine, no statistically significant correlation was found between hs-CRP levels and WMHs in these patients.

Beyond migraine, WMHs have also been associated with advancing age, vascular risk factors, and inflammatory conditions. In the context of migraines, previous studies have demonstrated a higher prevalence of these lesions among affected patients. However, their underlying pathophysiological mechanisms remain insufficiently elucidated. WMHs may represent imaging markers of focal cerebral hypoperfusion related to migraine attacks, potentially resulting from recurrent impairment of small penetrating arteries and subsequent minor ischemic brain injury. Although migraine is associated with WMHs, these alterations may not be clinically relevant or may reflect pathophysiological mechanisms different from those underlying vascular lesions that lead to cognitive decline. While neuroimaging results indicated structural or functional alterations, no corresponding cognitive impairment was detected in the migraine group based on the administered test battery.

Regarding the genetic component, neither the NOS1 nor the NOS3 polymorphisms analyzed in these studies showed a clear association with migraine risk. The absence of an association for this specific SNP does not preclude a potential role of other NOS3 gene polymorphisms in susceptibility to this condition. Available data suggest that syncope in patients with migraine, particularly those with aura, may reflect an underlying component of vascular and endothelial dysfunction, with a possible genetic contribution from the rs11172113 variant in LRP1.

Women with MA, particularly those with high attack frequency, long disease duration, or additional CVRFs, may represent the subgroup most warranting careful vascular risk assessment. This scoping review suggests a fundamental paradox: while macrovascular structure appears largely preserved, microvascular dysfunction, elevated inflammatory biomarkers, increased WMH burden, and independent IS risk appear to coexist, defining a distinct and clinically relevant vascular phenotype.

From a clinical standpoint, current evidence supports integrating aura status as an independent risk modifier into CV risk stratification, as traditional scores such as SCORE2 and Framingham may underestimate risk in this population. PFO screening may be considered in young patients with cryptogenic ischemic events; periodic inflammatory biomarker surveillance may be warranted in women with MA; COC may require careful risk–benefit assessment in women under 45; while triptans need not necessarily be withheld on cardiovascular grounds absent specific contraindications.

Whether neuroimaging surveillance for WMH progression warrants broader implementation remains to be established. The roles of NOS polymorphisms, direct causal links between endothelial dysfunction and migraine-specific vascular events, and cardioembolic mechanisms underlying migraine-associated stroke remain speculative, and future prospective studies should prioritize elucidating these causal pathways and developing migraine-specific risk frameworks.

Ultimately, the ‘healthy vessel’ phenotype should not be misinterpreted as CV protection, but may reflect the complexity of migraine’s vascular biology and the potential limitations of conventional risk stratification in this population.

A particularly critical gap identified across all seven thematic domains concerns chronic migraine. Only one included study explicitly stratified outcomes by CM status [30]. All remaining domains addressed broader migraine subtypes without CM-specific data. Future research must prioritize dedicated CM cohorts, and until such evidence is available, clinicians should apply vigilant cardiovascular monitoring strategies in CM patients.

Future research should prioritize (1) sex-stratified prospective cohort studies with granular aura characterization (frequency, duration, type) and longitudinal CV event adjudication, to establish causal pathways and quantify attributable risk; (2) studies directly measuring cerebral, rather than systemic, endothelial function during both ictal and interictal phases, to resolve the discrepancy between null systemic findings and elevated cerebrovascular risk; (3) large-scale genetic studies in multiethnic populations to replicate the LRP1 syncope association and identify additional vascular susceptibility loci; and (4) prospective validation of the MARS+ score as a clinical risk stratification tool in migraine populations beyond the initial derivation cohort.

## Figures and Tables

**Table 1:** Cardiovascular risk assessment.

Author Year	Study Design	Sample Size	Migraine Subtype	Assessment Method	Main Findings	Interpretation
Al-Hassany et al. [18]	Cross-sectional.	1085 M/6181 C.	Active, History, MA.	CVRFs (Smoking, BMI, BP, etc.).	Females: Smoking OR 0.72; Diabetes OR 0.74; High DBP OR 1.16.	CVRFs inversely related to M in females (except DBP).
Al-Hassany et al. [19] (Lifelines/JAMA)	Prospective cohort.	28,139 M/115,000 C.	Prevalent, Incident.	SCORE2 CV risk score.	High risk (SCORE2 ≥ 10%): Prevalent M OR 0.43; Incident M OR 0.17.	Higher CV risk associated with lower migraine odds.
Cloet et al. [20]	Case–control.	225 M/421 C.	MWA, MwoA.	CSVD on MRI/CT.	MWA vs. C for Grade S1 CSVD: OR 0.35 (*p* = 0.048). Adjusted: NS.	MWA not independently associated with CSVD.
Gardener et al. [21]	Cross-sectional.	273 M/1065 C.	MA, MO.	Hypertension (HTN).	HTN and M: OR 1.76. Whites OR 2.42; Hispanics OR 1.76.	Strong M-HTN association, especially if long-duration.
Goulart et al. [22]	Cross-sectional.	4411 M/10,542 C.	Definite, Probable.	HTN, Lipids, MetS (MS).	Men: HTN OR 0.53. Women: Dyslipidemia OR 1.25.	Gender-specific associations: inverse HTN/MS in men; positive lipids in women.
Huang et al. [23]	Prospective cohort.	11,743 M/254,051 C.	Clinical/Self-rep.	ASCVD (CHD, Stroke, PAD).	Total ASCVD HR 1.14 (*p* < 0.001); PAD HR 1.23 (*p* = 0.011).	M elevates long-term ASCVD risk independently.
Ibrahimi et al. [24]	Cohort (Women).	5612 M/21,927 C.	Active, History, Incid.	Framingham (FRS).	History M: FRS ≥ 10% OR 1.76. Active M: OR 0.64.	High FRS only in past M; low FRS in active/incident M.
Ihara et al. [25]	Retrospective.	5535 CHC/21,520 no.	MwA, MwoA.	AIS, AMI, DVT/PE.	Non-CHC users: MwA vs. MwoA AIS HR 2.45 (*p* < 0.001).	MwA has higher vascular risk than MwoA in non-CHC users.
Kurth et al. [26]	Prospective cohort.	3612 M/24,246 C.	MWA, MwoA.	Major CVD (MI, Stroke).	MWA CVD rate: 3.36/1000 PY vs. 2.11/1000 in MWoA (*p* < 0.001).	MWA is a major CVD risk factor in women.
Lipton et al. [27]	Cross-sectional.	6723 M.	Episodic.	FRS score.	≥1 CV risk factors: 69.5% women, 74.3% men.	CV risk increases with age; consider triptan risk/benefit.
Monteith et al. [28]	Prospective cohort.	262 M/1030 C.	MA, MwoA.	Stroke, MI.	Overall: No association. Smokers with M: Stroke HR 3.17.	Smoking modifies M–stroke relationship in older adults.
Peles et al. [29]	Retrospective.	26,054 M.	M (general).	Triptan vs. CV events.	90-day post-triptan CV events: 0.3%. aHR 0.96 (NS).	Triptan use not associated with increased CV risk.
Tekgol Uzuner et al. [30]	Retrospective.	2712 M.	MwoA, MwA, CM.	HTN, DM, CAD.	HTN in <30 y: CM 47.2% vs. MwoA 23.7% (*p* < 0.001).	CM has significantly higher CVRFs across all ages.
Yang et al. [31]	Prospective cohort.	436 M/3019 C.	MA, MwoA.	FRS, Glucose, Lipids.	Baseline FRS 0.06; Glucose 6.44 mmol/L.	Tracks M features and CVD risk in Asian women.

Abbreviations: M = migraine patients; C = controls; MA = migraine with aura; MO = migraine without aura; MWA/MwA = migraine with aura; MWoA/MwoA = migraine without aura; CM = chronic migraine; OR = odds ratio; HR = hazard ratio; aHR = adjusted hazard ratio; NS = not significant; CVRF(s) = cardiovascular risk factor(s); BMI = body mass index; BP = blood pressure; DBP = diastolic blood pressure; HTN = hypertension; DM = diabetes mellitus; MetS = metabolic syndrome; CAD = coronary artery disease; ASCVD = atherosclerotic cardiovascular disease; CHD = coronary heart disease; PAD = peripheral artery disease; MI = myocardial infarction; CVD = cardiovascular disease; CV = cardiovascular; SCORE2 = Systematic COronary Risk Evaluation 2; FRS = Framingham Risk Score; AIS = acute ischemic stroke; AMI = acute myocardial infarction; DVT = deep vein thrombosis; PE = pulmonary embolism; CHC = combined hormonal contraceptives; PY = person-years; CSVD = cerebral small vessel disease; MRI = magnetic resonance imaging; CT = computed tomography.

**Table 2:** Stroke risk and outcomes.

Author Year	Study Design	Sample Size	Migraine Subtype	Assessment Method	Main Findings	Interpretation
Androulakis et al. [32]	Prospective cohort.	1622 M/11,136 C.	MA, MO.	Ischemic stroke.	MA vs. C: HR 1.7. Cardioembolic HR 3.7.	MA in late middle age increases cardioembolic stroke risk.
Androulakis et al. [33]	Prospective cohort.	1575 M/10,017 C.	MA (early/late onset).	Ischemic stroke.	Late-onset MA (≥50 y): HR 2.17. Early-onset: NS.	Late-onset MA is a stronger predictor for stroke/mortality.
De Giuli et al. [34]	Cohort.	1738 Stroke pts.	MA, MwoA.	AF, PFO.	MA with PFO: OR 2.92. Women with MA + PFO: OR 8.23.	MA + PFO strongly linked to brain ischemia.
Gill et al. [35]	Retrospective.	1.19 M patients.	MA, Risk categories.	19 vascular events.	Ischemic Stroke rate/1000 PY: MA 8.6 vs. Low-risk M 2.9.	MA and high-risk M patients have highest event rates.
Gollion et al. [36]	Cross-sectional.	144 M/271 C.	MWA, MwoA.	Atherosclerosis (LAA).	LAA any grade: M 24.3% vs. C 50.9% (OR 0.44, *p* = 0.005).	M negatively associated with large artery atherosclerosis.
Kurth et al. [37]	Prospective cohort.	17,531 M/98,010 C.	M (general).	Major CVD, stroke.	M vs. C: Stroke HR 1.62 (*p* < 0.01); CV Mortality HR 1.37.	M consistently linked with increased ischemic stroke risk.
Kuybu et al. [38]	Retrospective.	834,875 M (young).	MA, MO.	Ischemic stroke (IS).	IS Prevalence: MA 3.7% vs. MO 1.2%. MA IS OR 3.23.	MA is an independent predictor of IS and AF in youth.
Lantz et al. [39]	Prospective twin study.	8635 M/44,769 C.	MA, Probable M.	Stroke.	MA: OR 1.27 (*p* = 0.05). Any M: NS.	Modest stroke risk increase specifically for MA.
Li et al. [40]	Cohort.	1810 pts.	MA, MO.	TIA, stroke.	Cryptogenic events: M OR 1.73; Cardioembolic: OR 2.00.	M strongly associated with cryptogenic and embolic events.
McCain et al. [41]	Prospective cohort.	1485 M.	MA, MO.	MARS+ Score.	MARS+ score ≥ 5: IS OR 4.09 (*p* < 0.001).	MARS+ score effectively predicts lifetime IS risk.
Sen et al. [42]	Longitudinal.	1516 M/9405 C.	MA, MO.	Atrial Fib (AF).	AF incidence: M 15% vs. C 17% (No significant difference).	AF may be a mediator but not uniquely frequent in M.
Velickovic et al. [43]	Retrospective.	12,448 M admissions.	MA, MO, Status M.	30-day readmission.	Aura: TIA HR 2.13. AIS HR 1.14 (NS).	Aura independently increases 30-day TIA readmission risk.
West et al. [44]	Retrospective.	712 AIS patients/34 M/34 C –PFO testing.	M, Frequent Aura.	PFO/RLS.	PFO prevalence: M 79% vs. C 59% (Gen. pop 18%).	Very high PFO prevalence in M patients with cryptogenic stroke.

Abbreviations: M = migraine patients; C = controls; MA = migraine with aura; MO = migraine without aura; MWA/MWoA = migraine with/without aura; OR = odds ratio; HR = hazard ratio; NS = not significant; IS = ischemic stroke; AIS = acute ischemic stroke; TIA = transient ischemic attack; AF = atrial fibrillation; PFO = patent foramen ovale; RLS = right-to-left shunt; LAA = large artery atherosclerosis; MARS = Migraine-Associated Risk Score; CV = cardiovascular; PY = person-years.

**Table 3:** Structural and functional vascular assessment.

Author Year	Study Design	Sample Size	Migraine Subtype	Assessment Method	Main Findings	Interpretation
Apelbaum et al. [45]	Cross-sectional.	4649 total.	MO, MA.	PWV (Stiffness).	PWV: NM 8.67; MA 8.11; MO 8.01 m/s (*p* > 0.05).	Aortic stiffness (PWV) is not associated with M.
Filippopulos et al. [46]	Cross-sectional.	337 M/1100 C.	MA, MO (pooled).	CACS (Calcification).	CACS: Men *p* = 0.41; Women *p* = 0.38 (NS).	M has no impact on coronary calcification.
Goulart et al. [47]	Cross-sectional.	383 M/2834 C.	MA, MO.	CAC score, C-IMT.	Adjusted: All associations NS. Crude: M had lower CAC.	M not independently associated with subclinical atherosclerosis.
Larsen et al. [48]	Population cohort.	428 M/3511 C.	MA, MwoA.	FMD (Endothelial).	Mean FMD: MA 5.08%, MWoA 5.31% (NS).	No relationship between endothelial function and M.
Linstra et al. [49]	Case–control.	594 M/199 C.	M (general).	Cold extremities.	Cold extremities: M vs. C OR 2.3 (*p* < 0.001).	M reported more cold extremities (vascular dysfunction).
van Os et al. [50]	Retrospective.	53 M/603 C	MA, MO.	CTA (Athero).	Intracranial Athero: M 51% vs. C 74% (adjRR 0.82).	M not associated with excess large vessel atherosclerosis.
Wen et al. [51]	Prospective cohort.	562 M/2708 C.	MWA, MwoA.	Retinal caliber.	Retinopathy OR 1.09 (*p* = 0.77). Calibers: NS.	No association between M and retinal microvasculature.
Wen et al. [52]	Prospective cohort.	279 M/1577 C.	Active, MA, MO.	ICAC (Carotid Calc.).	ICAC volume: M vs. C difference −0.19 (*p* < 0.001).	M patients had **less** intracranial carotid calcification.

Abbreviations: M = migraine patients; C = controls; MA = migraine with aura; MO = migraine without aura; MWA/MWoA = migraine with/without aura; NM = non-migraine; OR = odds ratio; NS = not significant; PWV = pulse wave velocity; FMD = flow-mediated dilation; CAC = coronary artery calcification; CACS = coronary artery calcification score; ICAC = intracranial carotid artery calcification; CTA = computed tomography angiography.

**Table 4:** Inflammatory and circulating biomarkers.

Author Year	Study Design	Sample Size	Migraine Subtype	Assessment Method	Main Findings	Interpretation
Avci et al. [12] (CRP)	Case–control.	216 M/216 C.	MO, MA.	hs-CRP.	M 1.94 ± 2.03 vs. C 0.82 ± 0.58 mg/L (*p* < 0.0001).	Elevated hs-CRP indicates a systemic proinflammatory state.
Tietjen et al. [53]	Population-based.	283 M/134 C.	MA, MO.	vWF, CRP, Fibrinogen.	Fibrinogen: M 316 vs. C 298 mg/dL (*p* = 0.007). CRP *p* = 0.03.	M (especially MA) linked to hypercoagulability/inflammation.

Abbreviations: M = migraine patients; C = controls; MA = migraine with aura; MO = migraine without aura; hs-CRP = high-sensitivity C-reactive protein; vWF = von Willebrand factor.

**Table 5:** Radiological and neuroimaging findings.

Author Year	Study Design	Sample Size	Migraine Subtype	Assessment Method	Main Findings	Interpretation
Avci et al. * [12] (WMH)	Case–control.	216 M/216 C.	MO, MA.	MRI (WMHs).	WMH: M 31.9% vs. C 9.7% (OR 4.35, *p* < 0.001).	Significantly higher WMH prevalence in M patients.
Vijiaratnam et al. [54]	Retrospective.	505 M.	MA, MwoA.	MRI, Doppler.	WMH: MA 39% vs. MWoA 41% (NS). Abnormal Doppler: 1 pt.	WMHs linked to age/CVRFs, not M subtype. Low imaging value.

Abbreviations: M = migraine patients; C = controls; MA = migraine with aura; MO/MWoA = migraine without aura; OR = odds ratio; NS = not significant; WMH(s) = white matter hyperintensity/hyperintensities; MRI = magnetic resonance imaging; CVRF(s) = cardiovascular risk factor(s). * Note: Avci et al. [12] appears in both Category 4 (hs-CRP findings) and Category 5 (WMH findings) as the study assessed two independent vascular outcomes with no significant correlation.

**Table 6:** Genetic and molecular biomarkers.

Author Year	Study Design	Sample Size	Migraine Subtype	Assessment Method	Main Findings	Interpretation
García-Martín et al. [55]	Case–control.	197 M/308 C.	MWA, MO.	NOS1 rs7977109.	Minor allele OR 0.94 (NS).	NOS1 variants not associated with migraine risk.
García-Martín et al. [56]	Case–control.	283 M/287 C.	MWA, MwoA.	NOS3 rs2070744.	Minor allele OR 0.91 (NS).	NOS3 SNP not associated with migraine risk.
Kuan et al. [57]	Cross-sectional.	1593 M.	MWA, MwoA.	LRP1 gene.	LRP1 rs11172113 & syncope: OR 4.00 (*p* = 0.045).	Genetic link between M susceptibility and vascular syncope.

Abbreviations: M = migraine patients; C = controls; MWA = migraine with aura; MO/MWoA = migraine without aura; OR = odds ratio; NS = not significant; NOS1 = neuronal nitric oxide synthase 1 (gene); NOS3 = endothelial nitric oxide synthase 3 (gene); NO = nitric oxide; SNP = single nucleotide polymorphism; LRP1 = low-density lipoprotein receptor-related protein 1.

**Table 7:** Neurocognitive profiles.

Author Year	Study Design	Sample Size	Migraine Subtype	Assessment Method	Main Findings	Interpretation
George et al. [13]	Prospective cohort.	2640 M/SNH/9955 C.	MA, M (general).	Incident dementia.	Dementia HR: M 1.04; MA 1.12 (both NS).	No association between M history and incident dementia.
Wen et al. [58]	Prospective cohort.	1309 M/5399 C.	Definite, MA, MO.	MMSE, cognitive-factor.	MMSE: M +0.21; MA +0.39 higher vs. C.	M patients (especially MA) score higher on cognitive tests.

Abbreviations: M = migraine patients; C = controls; MA = migraine with aura; MO = migraine without aura; HR = hazard ratio; NS = not significant; MMSE = Mini-Mental State Examination.

## Data Availability

No new data were generated or analyzed during this study. This scoping review is based entirely on previously published literature, which is cited accordingly throughout the manuscript.

## Supplementary Materials

The following supporting information can be downloaded at: https://www.mdpi.com/article/10.3390/life16060900/s1. Supplementary File S1: Table S1: Category 1: Cardiovascular Risk Assessment (14 studies); Table S2: Category 2: Stroke Risk and Outcomes (13 studies); Table S3: Category 3: Structural and Functional Vascular Assessment (8 studies); Table S4: Category 4: Inflammatory and Circulating Biomarkers (2 studies); Table S5: Category 5: Radiological and Neuroimaging Findings (2 studies); Table S6: Category 6: Genetic and Molecular Biomarkers (3 studies); Table S7: Category 7: Neurocognitive Profiles (2 studies). Supplementary File S2: Table S8: PRISMA-ScR Checklist—Preferred Reporting Items for Systematic Reviews and Meta-Analyses Extension for Scoping Reviews. The study selection process is illustrated in the PRISMA flow diagram presented in Section 3 (Results).

## Abbreviations

The following abbreviations are used in this manuscript:

ADMA	Asymmetric dimethylarginine
AF	Atrial fibrillation
aHR	Adjusted hazard ratio
AIS	Acute ischemic stroke
AMI	Acute myocardial infarction
AMPP	American Migraine Prevalence and Prevention
ARIC	Atherosclerosis risk in communities
ASCVD	Atherosclerotic cardiovascular disease
BMI	Body mass index
BP	Blood pressure
C	Control (group)
CAC	Coronary artery calcification
CACSAC	Coronary Artery Calcification Score
CADASIL	Cerebral autosomal dominant arteriopathy with subcortical infarcts and leukoencephalopathy
CAD	Coronary artery disease
CAMERA	Cerebral Abnormalities in Migraine, an Epidemiological Risk Analysis (Study)
CHC	Combined hormonal contraceptives
CHD	Coronary heart disease
CI	Confidence interval
cIMT	Carotid intima-media thickness
CM	Chronic migraine
CSVD	Cerebral small vessel disease
CT	Computed tomography
CTA	Computed tomography angiography
CV	Cardiovascular
CVD	Cardiovascular disease
CVRF(s)	Cardiovascular risk factor(s)
DALYs	Disability-adjusted life years
DBP	Diastolic blood pressure
DM	Diabetes mellitus
DUST	Dutch Study on the Genetics of Migraine (Investigators)
DVT	Deep vein thrombosis
ELSA-Brasil	Longitudinal Study of Adult Health—Brazil
eNOS	Endothelial nitric oxide synthase
EPC(s)	Endothelial progenitor cell(s)
FMD	Flow-mediated dilation
FRS	Framingham Risk Score
HR	Hazard ratio
hs-CRP	High-Sensitivity C-Reactive Protein
HTN	Hypertension
ICAC	Intracranial carotid artery calcification
ICAM-1	Intercellular Adhesion Molecule-1
ICHD	International Classification of Headache Disorders
IS	Ischemic stroke
LAA	Large artery atherosclerosis
LRP1	Low-Density Lipoprotein Receptor-Related Protein 1
M	Migraine (patients)
MA	Migraine with aura
MARS	Migraine-Associated Risk Score
MECH-HK	Migraine Exposures and Cardiovascular Health in Hong Kong (Cohort)
MEDLINE	Medical Literature Analysis and Retrieval System Online
MetS	Metabolic syndrome
MI	Myocardial infarction
MMSE	Mini-Mental State Examination
MO	Migraine without aura
MRA	Magnetic resonance angiography
MRI	Magnetic resonance imaging
MWA/MwA	Migraine with Aura
MWoA/MwoA	Migraine without Aura
NHS II	Nurses’ Health Study II
NM	Non-migraine/no migraine
NO	Nitric oxide
NOS1	Neuronal Nitric Oxide Synthase 1 (gene)
NOS3	Endothelial Nitric Oxide Synthase 3 (gene)
NS	Not significant
OR	Odds ratio
OSF	Open science framework
PAD	Peripheral artery disease
PCC	Population, concept, context (framework)
PE	Pulmonary embolism
PFO	Patent Foramen Ovale
PRISMA-ScR	Preferred Reporting Items for Systematic Reviews and Meta-Analyses extension for Scoping Reviews
PTX3	Pentraxin-3
PubMed	Public Medical Database (NCBI)
PWV	Pulse wave velocity
PY	Person-years
RCT	Randomized controlled trial
RLS	Right-to-left shunt
SBP	Systolic blood pressure
SCORE2	Systematic COronary Risk Evaluation 2
SNP	Single nucleotide polymorphism
TIA	Transient ischemic attack
VCAM-1	Vascular Cell Adhesion Molecule-1
VEGF	Vascular Endothelial Growth Factor
vWF	von Willebrand factor
WMH(s)	White matter hyperintensity/hyperintensities

## References

[1] Dong L., Dong W., Jin Y., Jiang Y., Li Z., Yu D. (2025). The global burden of migraine: A 30-year trend review and future projections by age, sex, country, and region. Pain Ther..

[2] Cole J.W., Kittner S.J. (2010). Meta-analysis of results from case control and cohort studies finds that migraine is associated with approximately twice the risk of ischaemic stroke. Evid.-Based Med..

[3] Schürks M., Rist P.M., Bigal M.E., Buring J.E., Lipton R.B., Kurth T. (2009). Migraine and cardiovascular disease: Systematic review and meta-analysis. BMJ.

[4] Zhao W., Wang D., Tan Y., Yang J., Zhang S. (2024). Migraine and the correlation between stroke: A systematic review and meta-analysis. Medicine.

[5] Nathan N., Ngo A., Khoromi S. (2024). Migraine and stroke: A scoping review. J. Clin. Med..

[6] Dzator J.S.A., Howe P.R.C., Wong R.H.X. (2021). Profiling cerebrovascular function in migraine: A systematic review and meta-analysis. J. Cereb. Blood Flow Metab..

[7] Davis D., Gregson J., Willeit P., Stephan B., Al-Shahi Salman R., Brayne C. (2012). Patent foramen ovale, ischemic stroke and migraine: Systematic review and stratified meta-analysis of association studies. Neuroepidemiology.

[8] Butt J.H., Franzmann U., Kruuse C. (2015). Endothelial function in migraine with aura—A systematic review. Headache.

[9] Tietjen G.E., Khubchandani J. (2015). Vascular biomarkers in migraine. Cephalalgia.

[10] Cuciureanu D.I., Bistriceanu C.E., Vulpoi G.A., Cuciureanu T., Antochi F., Roceanu A.M. (2024). Migraine comorbidities. Life.

[11] Demaerschalk B.M. (2011). Migraine is associated with an increased risk of cervicocephalic arterial dissection. Neurology.

[12] Avci A.Y., Lakadamyali H., Arikan S., Benli U.S., Kilinc M. (2015). High sensitivity C-reactive protein and cerebral white matter hyperintensities on magnetic resonance imaging in migraine patients. J. Headache Pain.

[13] George K.M., Folsom A.R., Sharrett A.R., Mosley T.H., Gottesman R.F., Hamedani A.G., Lutsey P.L. (2020). Migraine headache and risk of dementia in the Atherosclerosis Risk in Communities Neurocognitive Study. Headache.

[14] Arksey H., O’Malley L. (2005). Scoping studies: Towards a methodological framework. Int. J. Soc. Res. Methodol..

[15] Levac D., Colquhoun H., O’Brien K.K. (2010). Scoping studies: Advancing the methodology. Implement. Sci..

[16] Tricco A.C., Lillie E., Zarin W., O’Brien K.K., Colquhoun H., Levac D., Moher D., Peters M.D.J., Horsley T., Weeks L. (2018). PRISMA Extension for Scoping Reviews (PRISMA-ScR): Checklist and explanation. Ann. Intern. Med..

[17] Headache Classification Committee of the International Headache Society (IHS) (2018). The International Classification of Headache Disorders, 3rd edition. Cephalalgia.

[18] Al-Hassany L., Acarsoy C., Ikram M.K., Bos D., MaassenVanDenBrink A. (2024). Sex-specific association of cardiovascular risk factors with migraine: The population-based Rotterdam Study. Neurology.

[19] Al-Hassany L., MaassenVanDenBrink A., Kurth T. (2024). Cardiovascular risk scores and migraine status. JAMA Netw. Open.

[20] Cloet F., Gueyraud G., Lerebours F., Munio M., Larrue V., Gollion C. (2024). Stroke due to small-vessel disease and migraine: A case-control study of a young adult with ischemic stroke population. Cephalalgia.

[21] Gardener H., Monteith T., Rundek T., Wright C.B., Elkind M.S., Sacco R.L. (2016). Hypertension and migraine in the Northern Manhattan Study. Ethn. Dis..

[22] Goulart A.C., Santos I.S., Lotufo P.A., Benseñor I.M. (2015). Gender aspects of the relationship between migraine and cardiovascular risk factors: A cross-sectional evaluation in the Brazilian Longitudinal Study of Adult Health (ELSA-Brasil). Cephalalgia.

[23] Huang Y., Yan W., Jia Y., Xie Q., Lei Y., Chen Z., Zhou Y., Xiao Z. (2025). Migraine and increased cardiovascular disease risk: Interaction with traditional risk factors and lifestyle factors. J. Headache Pain.

[24] Ibrahimi K., Rist P.M., Carpenet C., Rohmann J.L., Buring J.E., Maassen van den Brink A., Kurth T. (2022). Vascular risk score and associations with past, current, or future migraine in women: Cohort study. Neurology.

[25] Ihara K., Pike C.W., Hui G., Gombar S., Jackson M.L., Callahan A., Tietjen G.E., Chiang C.C. (2025). Estrogen exposure from modern contraceptives and vascular risk in women with migraine: A nationwide electronic medical record database study. Cephalalgia.

[26] Kurth T., Rist P.M., Ridker P.M., Kotler G., Bubes V., Buring J.E. (2020). Association of migraine with aura and other risk factors with incident cardiovascular disease in women. JAMA.

[27] Lipton R.B., Reed M.L., Kurth T., Fanning K.M., Buse D.C. (2017). Framingham-based cardiovascular risk estimates among people with episodic migraine in the US population: Results from the American Migraine Prevalence and Prevention (AMPP) Study. Headache.

[28] Monteith T.S., Gardener H., Rundek T., Elkind M.S., Sacco R.L. (2015). Migraine and risk of stroke in older adults: Northern Manhattan Study. Neurology.

[29] Peles I., Shneyour R.S., Levanon E., Steen Y.M., Abu Salameh I., Gordon M., Abuhasira R., Novack V., Ifergane G. (2025). Cardiovascular risk and triptan usage among patients with migraine. Headache.

[30] Tekgol Uzuner G., Yalin O.O., Uluduz D., Ozge A., Uzuner N. (2021). Migraine and cardiovascular risk factors: A clinic-based study. Clin. Neurol. Neurosurg..

[31] Yang Q., Sun Q., Loke A.Y., Wang H.H., Qin J., Yang L., Xie Y.J. (2024). Cohort profile: Migraine exposures and cardiovascular health in Hong Kong Chinese women (MECH-HK). BMJ Open.

[32] Androulakis X.M., Kodumuri N., Giamberardino L.D., Rosamond W.D., Gottesman R.F., Yim E., Sen S. (2016). Ischemic stroke subtypes and migraine with visual aura in the ARIC study. Neurology.

[33] Androulakis X.M., Sen S., Kodumuri N., Zhang T., Grego J., Rosamond W., Gottesman R.F., Shahar E., Peterlin B.L. (2019). Migraine age of onset and association with ischemic stroke in late life: 20 years follow-up in ARIC. Headache.

[34] De Giuli V., Grassi M., Locatelli M., Gamba M., Morotti A., Bonacina S., Mazzoleni V., Pezzini D., Magoni M., Monastero R. (2021). Cardiac sources of cerebral embolism in people with migraine. Eur. J. Neurol..

[35] Gill K., Chia V.M., Hernandez R.K., Navetta M. (2020). Rates of vascular events in patients with migraine: A MarketScan® database retrospective cohort study. Headache.

[36] Gollion C., Guidolin B., Lerebours F., Rousseau V., Barbieux-Guillot M., Larrue V. (2022). Migraine and large artery atherosclerosis in young adults with ischemic stroke. Headache.

[37] Kurth T., Winter A.C., Eliassen A.H., Dushkes R., Mukamal K.J., Rimm E.B., Willett W.C., Manson J.E., Rexrode K.M. (2016). Migraine and risk of cardiovascular disease in women: Prospective cohort study. BMJ.

[38] Kuybu O., Amireh A., Davis D., Kelley R.E., Javalkar V. (2020). Prevalence of ischemic stroke and atrial fibrillation in young patients with migraine national inpatient sample analysis. J. Stroke Cerebrovasc. Dis..

[39] Lantz M., Sieurin J., Sjolander A., Waldenlind E., Sjostrand C., Wirdefeldt K. (2017). Migraine and risk of stroke: A national population-based twin study. Brain.

[40] Li L., Schulz U.G., Kuker W., Rothwell P.M. (2015). Age-specific association of migraine with cryptogenic TIA and stroke: Population-based study. Neurology.

[41] McCain C.R., Parrish M.H., Melikov P., Rosamond W.D., Sengupta S., Spinale F., Trivedi T., Wood S., Sen S. (2025). Introducing a risk score for predicting ischemic stroke in migraine with or without visual aura. Cephalalgia.

[42] Sen S., Androulakis X.M., Duda V., Alonso A., Chen L.Y., Soliman E.Z., Magnani J., Trivedi T., Merchant A.T., Gottesman R.F. (2018). Migraine with visual aura is a risk factor for incident atrial fibrillation: A cohort study. Neurology.

[43] Velickovic Ostojic L., Liang J.W., Sheikh H.U., Dhamoon M.S. (2018). Impact of aura and status migrainosus on readmissions for vascular events after migraine admission. Headache.

[44] West B.H., Noureddin N., Mamzhi Y., Low C.G., Coluzzi A.C., Shih E.J., Gevorgyan Fleming R., Saver J.L., Liebeskind D.S., Charles A. (2018). Frequency of patent foramen ovale and migraine in patients with cryptogenic stroke. Stroke.

[45] Apelbaum P.N., Goulart A.C., Santos I.S., Lotufo P.A., Baena C.P., Benseñor I.J.M. (2020). Migraine and arterial stiffness in the Brazilian Longitudinal Study of Adult Health: ELSA-Brasil. Am. J. Hypertens..

[46] Filippopulos F.M., Schoeberl F., Becker H.C., Becker-Bense S., Eren O., Straube A., Becker A. (2019). Coronary artery calcification score in migraine patients. Sci. Rep..

[47] Goulart A.C., Santos I.S., Bittencourt M.S., Lotufo P.A., Benseñor I.M. (2016). Migraine and subclinical atherosclerosis in the Brazilian Longitudinal Study of Adult Health (ELSA-Brasil). Cephalalgia.

[48] Larsen J.S., Skaug E.A., Wisloff U., Ellingsen O., Stovner L.J., Linde M., Hagen K. (2016). Migraine and endothelial function: The HUNT3 Study. Cephalalgia.

[49] Linstra K.M., Perenboom M.J.L., van Zwet E.W., van Welie F.C., Fronczek R., Tannemaat M.R., Wermer M.J.H., Maassenvandenbrink A., Terwindt G.M. (2020). Cold extremities in migraine: A marker for vascular dysfunction in women. Eur. J. Neurol..

[50] van Os H.J.A., Mulder I.A., Broersen A., Algra A., van der Schaaf I.C., Kappelle L.J., Velthuis B.K., Terwindt G.M., Schonewille W.J., Visser M.C. (2017). Migraine and cerebrovascular atherosclerosis in patients with ischemic stroke. Stroke.

[51] Wen K.X., Mutlu U., Ikram M.K., Kavousi M., Klaver C.C., Tiemeier H., Franco O.H., Ikram M.A. (2018). The retinal microcirculation in migraine: The Rotterdam Study. Cephalalgia.

[52] Wen K.X., Ikram M.A., Franco O.H., Vernooij M., MaassenVanDenBrink A., Bos D., Kavousi M. (2019). Association of migraine with calcification in major vessel beds: The Rotterdam Study. Cephalalgia.

[53] Tietjen G.E., Khubchandani J., Herial N., Palm-Meinders I.H., Koppen H., Terwindt G.M., van Buchem M.A., Launer L.J., Ferrari M.D., Kruit M.C. (2018). Migraine and vascular disease biomarkers: A population-based case-control study. Cephalalgia.

[54] Vijiaratnam N., Barber D., Lim K.Z., Paul E., Jiang M., Chosich B., Wijeratne T. (2016). Migraine: Does aura require investigation?. Clin. Neurol. Neurosurg..

[55] García-Martín E., Martínez C., Serrador M., Alonso-Navarro H., Navacerrada F., García-Albea E., Agúndez J.A.G., Jiménez-Jiménez F.J. (2015). Neuronal nitric oxide synthase (nNOS, NOS1) rs693534 and rs7977109 variants and risk for migraine. Headache.

[56] García-Martín E., Navarro-Muñoz S., Rodriguez C., Serrador M., Alonso-Navarro H., Calleja M., Turpín-Fenoll L., Recio-Bermejo M., García-Ruiz R., Millán-Pascual J. (2019). Association between endothelial nitric oxide synthase (NOS3) rs2070744 and the risk for migraine. Pharmacogenom. J..

[57] Kuan A.S., Chen S.P., Wang Y.F., Fuh J.L., Cheng C.Y., Peng K.P., Wang S.J. (2019). Risk factors and psychological impact of syncope in migraine patients. Cephalalgia.

[58] Wen K., Nguyen N.T., Hofman A., Ikram M.A., Franco O.H. (2016). Migraine is associated with better cognition in the middle-aged and elderly: The Rotterdam Study. Eur. J. Neurol..

[59] Pang Y., Cao T., Zhang Q., Hu H., Wang Z., Nie J., Jin M., Chen G., Zhang X. (2023). Retinal microvasculature features in patients with migraine: A systematic review and meta-analysis. Front. Neurol..

