# Supplementary material for: Migraine and Cardiovascular Risk: A Scoping Review of Vascular Outcomes, Risk Assessment, and Endothelial Dysfunction
Source: Life (Basel). 2026 May 27;16(6):900. doi: 10.3390/life16060900 (PMC13302511; doi:10.3390/life16060900)
Supplement: Supplementary file 1 [file life-16-00900-s001.zip › Supplementary File S1.pdf]

## Supplementary File S1

**Table S1. Category 1: Cardiovascular Risk Assessment (14 studies)**

| Author Year | Study Design | Sample Size (N migraine/N controls) | Migraine Subtype | Assessment Method | Main Findings (Exact Numbers) | Interpretation |
|---|---|---|---|---|---|---|
| **Al-Hassany 2024 (Rotterdam Study)** | Cross-sectional cohort. | 1085 migraine/6181 no migraine (total N = 7266). | Active migraine (n = 422, 38.9%), History of migraine (n = 663, 61.1%), MA (n = 221, 20.4%). | Traditional cardiovascular risk factors: smoking, obesity, hypercholesterolemia, hypertension, diabetes mellitus, blood pressure, lipid levels, BMI, fasting glucose. | Females: Smoking OR 0.72 (0.58–0.90); Diabetes OR 0.74 (0.56–0.98); Fasting glucose OR 0.90 (0.82–0.98) - lower migraine prevalence; Higher diastolic BP OR 1.16 (1.04–1.29) – higher migraine prevalence. Males: No associations. | Traditional CVRFs inversely related to migraine in females only; increased DBP suggests microvascular dysfunction. |
| **Al-Hassany et al., 2024 (Lifelines Cohort)** | Prospective population-based cohort. | 25,915 prevalent migraine/2224 incident migraine/115,000 without migraine (total N = 140,915). | Prevalent migraine (at baseline), Incident migraine (during follow-up). | SCORE2 cardiovascular risk score (age, cholesterol, smoking status, diabetes, systolic blood pressure). | SCORE2 ≥10% vs. <1%: Prevalent migraine OR 0.43 (0.39–0.48) vs. 1; Incident migraine OR 0.17 (0.10–0.27) vs. 1. Inverse dose–response. | Higher CV risk associated with lower migraine odds; healthy vascular system increases migraine probability. |
| **Cloet et al., 2024** | Retrospective case–control. | 115 MWA/110 MWoA/421 no migraine (total N = 646, ages 18–54 years, first ischemic stroke). | Migraine with aura (MWA), Migraine without aura (MWoA). | Cerebral small vessel disease (CSVD) on MRI/CT, graded as S0 (no CSVD), S1 (potentially causal), S2 (uncertain causality), S3 (unlikely causal). | Univariate: MWA vs. no migraine for grade S1 CSVD OR 0.35 (p = 0.048); CSVD of any grade significantly less frequent in migraine (p = 0.003), MWoA (p = 0.04), MWA (p = 0.02). Multivariate adjusted: Grade S1 CSVD with MWA OR 0.72 (p = 0.56, NS); Any grade CSVD with migraine OR 0.78 (p = 0.34, NS). | MWA NOT associated with CSVD. Results invalidate hypothesis of increased CSVD-related stroke risk in MWA population. |
| **Gardener et al., 2016** | Cross-sectional cohort. | 273 migraine (78 MA, 195 MO)/1065 no migraine (total N = 1338). | MA: visual aura (spots, stars, lines, flashing lights); MO: 4–72 h attacks, unilateral, pulsating, nausea/photophobia/phonophobia. | Hypertension (BP ≥140/90 mmHg, self-report, or antihypertensive medication use); Duration (≤9 vs. >9 years) and control (BP < 140/90) assessed. | Hypertension and migraine: OR 1.76. Systolic BP: Migraine 147 ± 20 mmHg vs. No migraine 144 ± 21 mmHg (p = 0.03). Fully-adjusted model 3 by race: Whites OR 2.42; Blacks OR 1.14; Hispanics OR 1.76. Long-duration hypertension: >2-fold increased odds of MA vs. no hypertension. | Strong association between hypertension and migraine (both MA and MO) in predominantly Hispanic older adults. Association particularly apparent for uncontrolled and long-duration hypertension. Suggests hypertension duration and control status important in migraine-vascular relationship. |
| **Goulart et al., 2015 (Second ELSA-Brasil Study)** | Cross-sectional (baseline ELSA-Brasil). | 4411 overall migraine 29.5% (22.5% women, 7.0% men)/10,542 no migraine (total N = 14,953). | Definite migraine (IHS 1.1 M O or 1.2 MA); Probable migraine (all criteria except one); Overall migraine category. | Cardiovascular risk factors: hypertension (SBP ≥ 140 or DBP ≥ 90 or medication), dyslipidemia, diabetes, metabolic syndrome (MS), smoking; Blood measurements (glucose, insulin, HbA1c, cholesterol, triglycerides). | Men: Hypertension OR 0.53 (0.36–0.79); MS OR 0.65 (0.43–0.99). Women: Dyslipidemia OR 1.25 (1.13–1.38). | Gender-specific associations: inverse hypertension/MS in men; positive dyslipidemia in women. |
| **Huang et al., 2025 (UK** | Prospective cohort (mean 12.9- | 11,743 migraine/254,051 no | Migraine: (i) documented clinical | Atherosclerotic cardiovascular | Multivariable-adjusted | Migraine elevates long-term |

| | | | | | | |
|---|---|---|---|---|---|---|
| **Biobank)** | year follow-up). | migraine (total N = 265,794). | diagnosis, or (ii) self-reported. | disease (ASCVD) and components: coronary heart disease (CHD, ICD-10 I20–I25), ischemic stroke (I63), peripheral artery disease (PAD, I70–I72, I73.9). | migraine vs. no migraine: Total ASCVD HR 1.12 (95% CI 1.05–1.20, PFDR < 0.001); All covariates model HR 1.14 (1.07–1.22, PFDR < 0.001); CHD HR 1.09 (1.01–1.18, PFDR = 0.023); Ischemic stroke HR 1.13 (0.95–1.34, p = 0.162, NS); PAD HR 1.23 (1.05–1.42, PFDR = 0.011). | ASCVD risk independent of acute treatments. |
| **Ibrahimi et al., 2022 (Women's Health Study)** | Cohort (female health professionals). | 27,539 total: 21,927 no migraine/1500 history of migraine/3579 active migraine at baseline/533 incident migraine during follow-up. | No migraine; History of migraine (past but not in year before enrollment); Active migraine at baseline; Incident migraine (first report during follow-up); Mixed MA and MO. | Framingham Risk Score (FRS) estimating 10-year CHD risk: age, total cholesterol, HDL, smoking, SBP (by antihypertensive treatment). Categories: ≤1% (≤12 points), 2–4% (13–16 points), 5–9% (17–19 points), ≥10% (≥20 points). | History of migraine: FRS ≥10% OR 1.76 (1.39–2.23). Active migraine: OR 0.64 (0.52–0.80). Incident: OR 0.42 (0.22–0.81). | High FRS only in past migraine; low FRS in active/incident migraine suggests healthy vascular system favors migraine. |
| **Ihara et al., 2022** | Retrospective cohort (electronic medical records, post-2010). | 5535 CHC (combined hormonal contraceptive) prescribed/21,520 CHC not prescribed; After propensity matching: MWA 1309 each group; MWoA 3417 each group. | Migraine with aura (MWA); Migraine without aura (MWoA); Female patients ages 18–45 with ≥3 visits in 3 years, ≥1 migraine-specific medication within 6 months. | Composite endpoint: acute ischemic stroke (AIS), acute myocardial infarction (AMI), deep vein thrombosis/pulmonary embolism (DVT/PE), IV thrombolytic administration. High-dimensional propensity score matching. | Overall vascular events: CHC users 2.06% (114/5535) vs. Non-users 2.54% (547/21,520). MWA vs. MWoA in non-CHC users (6201 matched pairs): AIS HR 2.45 (95% CI 1.58–3.78, p < 0.001); Composite HR 1.34 (1.08–1.67, p = 0.008). | CHC as not associated with a significant increase in vascular risk in women aged 18–45 years with migraine, however, in those who never received CHC, MWA as associated with higher vascular risks compared to MWoA. |
| **Kurth et al., 2020** | Prospective cohort study (Women's Health Study). | 1435 migraine with aura/2177 migraine without aura/24,246 no migraine (total N = 27,858 women). | Migraine with aura (MWA); Migraine without aura (MWoA) or no migraine (reference group). | Major CVD (myocardial infarction, stroke, or CVD death); cardiovascular outcomes self-reported and confirmed by endpoint committee of physicians; nonfatal stroke confirmed if new focal neurologic deficit >24 h; MI confirmed using WHO criteria. | Adjusted Incidence Rates for Major CVD: The adjusted incidence rate of major CVD per 1000 person-years was 3.36 (95% CI, 2.72–3.99) for women with migraine with aura, significantly higher than 2.11 (95% CI, 1.98–2.24) for women with migraine without aura or no migraine (P < .001). | Migraine with aura is a significant risk factor for CVD in women, comparable to or exceeding the impact of several traditional vascular risk factors. |
| **Lipton et al., 2017** | Cross-sectional analysis: AMPP Study questionnaire. | 5227 women/1496 men. | Episodic migraine. | CVD event risk was calculated using the nonlaboratory Framingham CV disease risk score (FRS). | >= 1 CV risk factor from the FRS: 69.5% women, 73.4% men; >=2 : 38.9% women, 41.6% men; >=3 : 18.6% women, 19.1% men. | Framingham estimated CV risk increases significantly with age – use triptans and ergot risk-to-benefit ratio. |
| **Monteith et al., 2015** | Population-based prospective cohort study. | 262 migraine (75 with aura, 187 without aura)/1030 no migraine. | Migraine with aura, migraine without aura, migraine (all types). | Combined vascular events (stroke, myocardial infarction, vascular death) assessed over mean follow-up of 11 years using Cox models; migraine diagnosis based on ICHD-II criteria via self-report questionnaire. | No association with migraine: Stroke HR 1.04 (0.64–1.70); MI HR 0.92 (0.52–1.63). Smokers with migraine: Stroke HR 3.17 (1.13–8.85). | Smoking modifies migraine-stroke relationship; no overall CV risk increase in older diverse cohort. |
| **Peles et al., 2025** | Retrospective, population-based cohort study. | 26,054 patients with migraine; 12,560 (48.2%) initiated triptan therapy, 13,494 (51.8%) did not use triptans. | Migraine (with and without aura not distinguished). | Cardiovascular events (myocardial infarction or ischemic stroke) within 90 days post-triptan purchase; triptan usage and CV risk factors | CV events in triptan users within 90 days: 0.3%. Triptan vs. non-triptan: aHR 0.96 (0.77–1.23), NS. | Triptan use not associated with increased CV risk, even with existing CV risk factors. |

| | | | | | | |
|---|---|---|---|---|---|---|
| | | | | identified using electronic medical records and ICD codes from January 2000 to January 2022. | | |
| **Tekgol Uzuner et al., 2021** | Retrospective study (Turkish Headache Database). | 2712 patients; 1868 (68.9%) migraine without aura (MWoA), 246 (9.1%) migraine with aura (MWA), 598 (22.1%) chronic migraine (CM). | Migraine without aura (MWoA), migraine with aura (MWA), chronic migraine (CM) (ICHD-III). | Vascular risk factors (hypertension, diabetes mellitus, coronary artery disease, hyperlipidemia) assessed in patients and first-degree relatives across three age groups (<30 years, 30–50 years, >50 years). | Under 30 years age group (patients): Hypertension: MWoA 23.7%, MWA 16.7%, CM 47.2%, p = 0.000173. Diabetes mellitus: MWoA 23.1%, MWA 47.9%, CM 47.9%, p = 0.000043. Coronary artery disease: MWoA 22.8%, MWA 13.3%, CM 47.2%, p = 0.000050. 30–50 years age group (patients): Hypertension: MWoA 52.2%, MWA 40.0%, CM 56.4%, p = 0.000120. Diabetes mellitus: MWoA 45.1%, MWA 5.1%, CM 13.8%, p = 0.002094. Coronary artery disease: MWoA 27.6%, MWA 28.0%, CM 41.2%, p = 0.011246. Over 50 years age group (patients): Hypertension: MWoA 56.2%, MWA 58.3%, CM 83.3%, p = 0.003701. Diabetes mellitus: MWoA 32.7%, MWA 55.6%, CM 65.5%, p = 0.004132. Coronary artery disease: MWoA 27.0%, MWA 22.2%, CM 63.0%, p = 0.001687. | Chronic migraine was associated with significantly higher prevalence of vascular risk factors (hypertension, diabetes mellitus, coronary artery disease) compared to episodic migraine with and without aura across all age groups. |
| **Yang et al. 2024 (MECH-HK Study)** | Longitudinal, prospective cohort study. | Total enrolled: 4221 Hong Kong Chinese women aged ≥30 years; Completed first follow-up: 3455 women (retention rate 81.9%); Migraine: 283; Probable migraine: 153; Non-migraine headaches: 128; Prevalence: 10.3% prevalence of migraine or probable migraine. | Migraine, probable migraine, migraine with aura (n = 44), migraine without aura (n = 156), migraine with and without aura (n = 83) (ICHD-3 criteria). | Migraine diagnosis following ICHD-3 criteria; migraine features longitudinally tracked using migraine diary and summarized by epidemiological metrics; cardiovascular health assessed using Framingham risk score (FRS); blood glucose and lipid levels measured via point-of-care testing. | Baseline: Mean FRS 0.06; Blood glucose 6.44 mmol/L. Follow-up (1.27 y): migraine attack frequency 0.99/month; Attack duration 7.70 h. | Longitudinal cohort tracking migraine features and CVD risk progression in Hong Kong women. |

**Table S2. Category 2: Stroke Risk and Outcomes (13 studies)**

| Author Year | Study Design | Sample Size (N migraine/N controls) | Migraine Subtype | Assessment Method | Main Findings (Exact Numbers) | Interpretation |
|---|---|---|---|---|---|---|
| **Androulakis et al., 2016 (ARIC Study)** | Prospective longitudinal cohort (20-year follow-up). | 1622 migraine/11,136 no headache (total N = 12,758). | Migraine with visual aura (MA), Migraine without visual aura (MO). | Adjudicated ischemic stroke and stroke subtypes: cardioembolic, lacunar, thrombotic. | MA vs. no headache: HR 1.7; Incidence rate 4.1 per 1000 person-years; Absolute risk 6% total population. MO vs. no headache: HR 1.2 (p = 0.28, NS). MA vs. MO adjusted: All | Migraine with visual aura in late middle age (mean age 59 years) associated with significantly increased risk of ischemic stroke, particularly cardioembolic subtype. Migraine without aura not |

| | | | | | | |
|---|---|---|---|---|---|---|
| | | | | | ischemic stroke HR 1.67 (p = 0.014); cardioembolic stroke HR 3.7 (p = 0.003); lacunar stroke HR 2.6 (p = 0.07, NS). | associated with stroke risk. Visual aura symptoms may relate to distal embolic mechanism. |
| **Androulakis et al., 2019 (ARIC Study)** | Prospective longitudinal cohort (20-year follow-up). | 447 MA/1128 MO from 11,592 total participants. | MA stratified by age of onset (<50 years vs. ≥50 years); MO. | Ischemic stroke incidence over 20 years. | MA onset ≥50 years (mean duration 4.75 years): Multivariable adjusted HR 2.17 (p < 0.001); Incidence rate 6.67 per 1000 person-years; absolute risk 8.27% (37/447).<br>MA onset < 50 years (mean duration 28.17 years): Multivariable adjusted HR 1.31 (p = 0.212, NS).<br>MO: No association with stroke regardless | MA late onset associated with ischemic stroke and CVD mortality, independent of vascular risk factors. |
| **De Giuli et al., 2020** | Single-center cohort (consecutive acute brain ischemia patients). | 1738 total patients (1017 men, 58.5%; mean age 67.9 ± 14.9 years) stratified by migraine status. | Migraine with aura (MA), Migraine without aura. | Atrial fibrillation (AF), Patent foramen ovale (PFO). | AF: No association between AF and history of migraine or pathogenic subtypes.<br>PFO entire cohort: OR 1.84; Age ≤ 55 years OR 2.21.<br>MA with PFO: Entire cohort OR 2.92; Age ≤ 55 years OR 2.92; Women OR 8.23. Cryptogenic stroke: MA prevalence in PFO carriers 17.9% vs. 6.2% in no PFO (p = 0.023). | Migraine not associated with increased AF risk.<br>MA strongly associated with brain ischemia in PFO carriers. Suggests alternative cardioembolic mechanism via paradoxical embolism in MA-related stroke. |
| **Gill et al., 2021** | Retrospective cohort. | 1195,696 migraine patients: MA (4.8%); Risk categories: high (2.8%), medium (15.5%), low (77.9%). | Migraine with aura (MA); Categorized into 4 vascular risk categories: MA; high, medium, low vascular risk. | Event rates (per 1000 person-years) for 19 vascular events overall, by risk category, by baseline characteristics. | Ischemic stroke rates (per 1000 PY): Overall 5.1; MA 8.6; high-risk 47.2; medium-risk 9.4; low-risk 2.9. | High-risk migraine patients had highest rates of all 19 vascular events.<br>MA patients had higher ischemic stroke incidence. |
| **Gollion et al., 2022** | Cross-sectional (young adults with first acute ischemic stroke). | 144 migraine (76 MWA, 68 MWoA)/271 no migraine (total N = 415, ages 18–54, mean 43.9 ± 8.7 years, 62.2% men). | Migraine with aura (MWA); Migraine without aura (MWoA). | Stenotic and nonstenotic large artery atherosclerosis (LAA) of extracranial/intracranial cerebral arteries evaluated and graded using ASCOD criteria (atherosclerosis, small-vessel disease, cardiac pathology, other causes, dissection). | Stenotic LAA: Migraine 6.9% vs. no migraine 15.5%, p < 0.001.<br>LAA any grade: Migraine 24.3% vs. no migraine 50.9%, p < 0.001.<br>Multivariable adjusted: Migraine and LAA any grade OR 0.44 (p = 0.005); MWoA OR 0.42 (p = 0.020); MWA OR 0.47 (p = 0.037). | Migraine had negative association with LAA independent of traditional vascular risk factors. |
| **Kurth et al., 2016 (Nurses' Health Study II)** | Prospective cohort study. | Total participants: 115,541 women; Women with migraine: 17,531 (15.2%); Women without migraine: 98,010. | Physician's diagnosis of migraine; Information on migraine aura, migraine frequency, or migraine specific drugs was not available. | Primary Outcome: Major cardiovascular disease, defined as a combined endpoint of myocardial infarction, stroke, or fatal cardiovascular disease.<br>Secondary Outcomes: Individual endpoints of myocardial infarction, stroke, angina/coronary revascularization procedures, and cardiovascular mortality. | Major cardiovascular disease (multivariable adjusted): Hazard ratio (HR) 1.50.<br>Stroke (multivariable adjusted): HR 1.62 (95% CI 1.37 to 1.92), P < 0.01.<br>Cardiovascular mortality (multivariable adjusted): HR 1.37, P = 0.04. | Migraine is consistently linked with an increased risk of ischemic stroke. |
| **Kuybu et al., 2020** | Retrospective cross-sectional | 834,875 young patients (aged | MA and MO. | Ischemic stroke (IS) was | Overall Prevalence of IS: 1.3%. | Migraine aura was identified as |

| | | | | | | |
|---|---|---|---|---|---|---|
| | study. | 18–44 years): MA (4.9%)/MO (95.1%). | | identified using International Classification of Diseases-9-CM codes. | Prevalence of IS in MA vs. MO: MA (3.7%) vs. MO (1.2%, P < 0.001). Independent Predictors of IS: Migraine with aura (OR 3.23, 95% CI 3.05–3.42, P < 0.001). | an independent predictor of both IS and AF in patients with a history of migraine. The presence of PVD confers a high risk of IS in young migraine patients. |
| **Lantz et al., 2017** | Prospective twin study. | Migraine with Aura: 3553 individuals. Non-aura Migraineous Headache: 5082 individuals. No Migraine: 44,769 individuals. Total Migraineous Headache: 8635 individuals. | Migraine with aura; Non-aura migraineous headache (including migraine without aura and probable migraine); Any migraineous headache. | Stroke Diagnoses: Obtained from national patient and cause of death registers, followed longitudinally from interview date until first stroke, death, or end of study on 31 December 2014. ICD-10 codes for cerebral ischemia (I63.0–I63.9 and G46.0–G46.8) and intracerebral hemorrhage (I61.0–I61.9) were used. | Migraine with aura: Stroke OR 1.27, P = 0.05. Any migraineous: Stroke OR 1.07, P = 0.39. | Overall migraine headache was not associated with an increased risk for stroke. However, there was a modestly increased risk for stroke specifically in patients with migraine with aura. |
| **Li et al., 2015** | Population-based cohort study. | 1810 eligible patients with TIA or ischemic stroke/668 (36.9%) had cryptogenic events, and 187 (28.0%) of those with cryptogenic events had a history of migraine. | Migraine with aura and migraine without aura. | Incident stroke (ischemic and hemorrhagic) over median follow-up of 22.3 years using hospital discharge codes and death certificates; migraine ascertained via standardized interview. | Migraine: Cryptogenic events OR 1.73, p < 0.0001; Cardioembolic stroke OR 2.00, p < 0.0001. MA: OR 1.78, p < 0.0001. MO: OR 2.05, p = 0.0003. | Migraine is most strongly associated with cryptogenic TIA and ischemic stroke. |
| **McCain et al., 2025** | Prospective evaluation of participants in the Atherosclerosis Risk in Communities Cohort (ARIC). | Initial: 15.792 Migraine: 1.485, among these, ischemic stroke: 112. | Migraine with visual aura, migraine without visual aura. | Traditional CV factors; Medications; Migraine Characteristics; Stroke Ascertainment. | MARS + Score: Migraine Associated Risk of Stroke Score: 0–21 points. MARS+ score of ≥5: 4.09 (95% confidence interval = 2.67–6.26, p < 0.001) for ischemic stroke. | MARS+ score of ≥5 indicates a significantly higher lifetime risk of ischemic stroke. |
| **Sen et al., 2018** | Longitudinal, community-based cohort study. | 1516 migraine (426 with visual aura, 1090 without visual aura)/9405 no headache. | Migraine with visual aura, migraine without visual aura (modified ICHD-3 criteria). | Incident atrial fibrillation over 20-year follow-up period; AF adjudicated using ECGs, discharge codes, and death certificates; migraine assessed in 1993–1995, followed through 2013. | AF incidence: 232 (15%) of 1516 with migraine developed AF; 1623 (17%) of 9405 without headache developed AF. | AF may be a potential mediator of migraine with visual aura–stroke risk. |
| **Velickovic et al., 2018** | Retrospective cohort study using Nationwide Readmissions Database. | 12,448 index admissions for migraine; 9972 (80.1%) women, mean age 45.5 ± 14.8 years; 3038 (24.41%) with aura, 1798 (14.44%) with status migrainosus. | Migraine with aura, migraine without aura, status migrainosus (ICD-9-CM codes). | 30-day readmission rates for acute ischemic stroke (AIS), transient ischemic attack (TIA), subarachnoid hemorrhage (SAH), and intracerebral hemorrhage (ICH) after index migraine admission; data from 2013 Nationwide Readmissions Database. | Aura associations (adjusted for age and vascular risk factors): TIA: HR 2.13 (95% CI 1.22–3.74), remained significant. AIS: HR 1.14 (95% CI 0.66–1.98, p = 0.64). Status migrainosus associations (adjusted models): AIS: HR 1.33 (95% CI 0.62–2.84, p = 0.47). TIA: HR 0.17 (95% CI 0.02–1.25, p = 0.08). | Migraine admission with aura was independently associated with TIA readmission within 30 days. |
| **West et al., 2018** | Retrospective, observational. | 68 cryptogenic stroke patients with adequate PFO testing and documented migraine history: 34 with migraine/34 without migraine (from total 712 ischemic stroke patients, 127 | Migraine (general), migraine with frequent aura (defined as aura in at least 50% of migraine attacks; n = 15, 44% of 34 migraine patients), migrainous infarction. | Migraine diagnosis identified by reviewing neurology notes in electronic medical record; Migraine with frequent aura defined as aura in ≥50% of attacks; PFO/RLS detection via | PFO prevalence by group: Cryptogenic stroke without migraine: 20/34 = 59%. Cryptogenic stroke with migraine: 27/34 = 79%. Cryptogenic stroke with | In patients with cryptogenic stroke who have migraine, there is a high prevalence (79%) of PFO. |

| | | | | | | |
|---|---|---|---|---|---|---|
| | | cryptogenic (18%)). | | positive bubble contrast study with transthoracic echocardiography (TTE), transesophageal echocardiography (TEE), or transcranial Doppler (TCD). | migraine with frequent aura: 14/15 = 93%. General population: 18%. | |

**Table S3. Category 3: Structural and Functional Vascular Assessment (8 studies)**

| Author Year | Study Design | Sample Size (N migraine/N controls) | Migraine Subtype | Assessment Method | Main Findings (Exact Numbers) | Interpretation |
|---|---|---|---|---|---|---|
| **Apelbaum et al., 2020** | Cross-sectional from ELSA-Brasil. | 4649 total: 2521 women (25.7% MO + 15% MA) + 2128 men (11% MO +4.3% MA). | MO + MA (mixed). | Arterial stiffness measured by pulse wave velocity (PWV): carotid-femoral (PWV-cf). | PWV-cf: NM 8.67 m/s; MA 8.11 m/s; MO 8.01 m/s. After adjusted models: NM, MA and MO: no difference ($p > 0.05$). | Aortic PWV was not associated with migraine. |
| **Filippopulos et al., 2019** | Cross-sectional. | 337 migraine/1100 no migraine (total N = 1437, similar CV risk profiles). | MA and MO (pooled analysis). | Coronary artery calcium score (CACS, Agatston score) by non-contrast enhanced computed tomography. | CACS migraine vs. no migraine: Men 188 ± 97 vs. 172 ± 88, p = 0.41; Women 109 ± 66 vs. 117 ± 70, p = 0.38 (p = 0.21, NS). | Migraine has no significant impact on coronary artery calcification development. |
| **Goulart et al., 2016 (ELSA-Brasil)** | Cross-sectional (subset of prospective cohort). | 383 definite migraine (163 MA, 220 MO)/2834 no migraine (total N = 3217). | Definite migraine meeting IHS codes 1.1 (MO) or 1.2 (MA). | Coronary artery calcium (CAC) score (continuous and categorized: 0/1–100/>100); Carotid intima-media thickness (C-IMT, continuous and dichotomized at 75th percentile: 0.68 mm). | CAC score = 0: MO 80.9% vs. MA 87.7% vs. No migraine 68.5% ($p < 0.001$ both). CAC 1–100: MO 15.5%, MA 10.4%, No migraine 20.7%. CAC >100: MO 3.6%, MA 1.8%, No migraine 10.8%. C-IMT mean ± SE: MO 0.574 ± 0.007 vs. MA 0.561 ± 0.008 vs. No migraine 0.612 ± 0.003 ($p < 0.001$ both). After multivariate adjustment: associations no longer significant | Despite aura symptoms, migraine NOT independently associated with subclinical atherosclerosis (CAC or C-IMT) in middle-aged Brazilian sample after adjusting factors. Migraineurs were younger, predominantly women, with lower CV risk factors. Strongest inverse crude association seen for CAC score. |
| **Larsen et al., 2016** | Population-based cohort study (HUNT3 Study). | Total participants: 3939. Any headache: 1673, Migraine: 428, MA: 182, MWA: 246, Tension-type headache (TTH): 933, Unclassified headache: 312. | Migraine with aura (MA), migraine without aura (MWA) (ICHD-2 criteria). | Endothelial function evaluated by Flow-mediated dilation (FMD) of brachial artery measured via high-resolution ultrasound. | Mean FMD: MA: 5.08% (4.46–5.69), MWA: 5.31% (4.81–5.81). | There was no relationship between FMD and migraine or other headache diagnoses. |
| **Linstra et al., 2020** | Case–control study. | 594 migraine patients/199 controls. | Migraine with or without aura. | Self-reported cold extremities as a marker for vascular dysfunction in migraine. Thermal discomfort and cold extremities (TDCE) and difficulties initiating sleep (DIS) were assessed using questionnaires. | TDCE migraineurs versus controls: OR 2.3, 95% confidence interval 1.4–3.7; $P < 0.001$ - after adjustment medication (OR 2.3, 95% CI 1.4–3.7; $P < 0.001$). DIS in Migraineurs vs. Controls: women OR 5.2, 95% CI 2.4–11.4; $P < 0.0001$; men OR 3.8; 95% CI 1.2–12.4; P = 0.02 | Women with migraine more often report cold extremities compared with controls. Female migraineurs with cold extremities experienced higher attack frequencies. |
| **Van Os et al., 2017** | Retrospective multicenter cohort study (DUST: Dutch Acute Stroke Study). | 53 with history of migraine (29 with aura)/603 no history of migraine. | Migraine with aura, migraine without aura (ICHD-II criteria). | Intra- and extracranial atherosclerotic changes on computed tomographic angiography; intracranial internal carotid artery | Atherosclerosis in intracranial vessels: Migraine 51% vs. No migraine 74%; Adjusted risk ratio 0.82. Atherosclerosis in extracranial | Migraine is not associated with excess atherosclerosis in large cerebral vessels among patients with acute ischemic stroke. |

| | | | | | | |
|---|---|---|---|---|---|---|
| | | | | calcification volumes on noncontrast computed tomography; patients aged 18–99 years with acute ischemic stroke. | vessels: Migraine 62% vs. No migraine 79%; Adjusted risk ratio 0.93. Internal carotid artery calcification volumes (largest vs. medium and smallest tertile): Migraine 23% vs. No migraine 35%; Adjusted risk ratio 0.93. | |
| **Wen et al., 2018** | Prospective population-based cohort study (Rotterdam Study). | 562 definite migraine/2708 no migraine. | Definite migraine, migraine with aura, migraine without aura, active migraine, non-active migraine (adapted ICHD-II criteria). | Retinal microvascular caliber (arteriolar and venular) and retinopathy assessed via fundus photography; participants aged 45+ years from 2006 cohort. | Retinopathy prevalence: No migraine 90:2618, Migraine 16:546. Model 2 (fully adjusted): OR 1.09 (95% CI 0.62–1.92, p = 0.77). Arteriolar caliber (mean difference): Model 3 (fully adjusted): -0.05 (95% CI -0.13; 0.03, p = 0.20). Venular caliber (mean difference, Model 3): Migraine -0.00 (95% CI -0.09; 0.08, p = 0.92). | No association was found between migraine or migraine with aura and retinopathy or retinal microvascular caliber. |
| **Wen et al., 2019 (Rotterdam Study - Arterial Calcification)** | Prospective population-based cohort. | 279 migraine (15%)/1577 no migraine (calculated from total N = 1856). | Migraine (lifetime), migraine with aura, migraine without aura, active migraine (<1 year since last attack), non-active migraine (>1 year since last attack). | Migraine questionnaire based on ICHD-II criteria modified from GEM study; 16- or 64-slice multidetector CT scanner (Somatom Sensation, Siemens) without contrast; Coronary artery calcification (CAC) via Agatston method; Intracranial internal carotid artery calcification (ICAC) via semi-automated method. | Primary outcome (ICAC): Model 2 (adjusted for all covariates): Difference in log-transformed ICAC volume in migraine vs. no migraine: -0.19 (95% CI -0.29, -0.08). | Persons with migraine had less arterial calcification in the intracranial carotid artery compared to those without migraine. |

**Table S4. Category 4: Inflammatory and Circulating Biomarkers (2 studies)**

| Author Year | Study Design | Sample Size (N migraine/N controls) | Migraine Subtype | Assessment Method | Main Findings (Exact Numbers) | Interpretation |
|---|---|---|---|---|---|---|
| **Avci et al., 2015 (hs-CRP findings)** | Case–control. | 216 migraine/216 controls (ages 18–50 years). | MO (n = 143), MA (n = 73) | High-sensitivity C-reactive protein (hs-CRP) measured by latex-enhanced immunoturbidimetry. | hs-CRP levels: Migraine patients 1.94 ± 2.03 mg/L vs. Control subjects 0.82 ± 0.58 mg/L (p ≤ 0.0001) | Significantly elevated hs-CRP levels in migraine patients indicate systemic proinflammatory state. |
| **Tietjen et al., 2018** | Population-based study (CAMERA 1 study - Cerebral Abnormalities in Migraine, an Epidemiologic Risk Analysis). | 283 migraine (155 with aura, 128 without aura)/134 controls with no severe headaches/total 417. | Migraine with aura (MA), migraine without aura (MO) (ICHD-2 criteria). | Plasma biomarkers of endothelial activation and vascular disease: fibrinogen, Factor II, D-dimer, high sensitivity C-reactive protein (hs-CRP), von Willebrand factor antigen (vWF Ag); samples collected ≥3 days after and >3 days before migraine attack. | Mean biomarker levels: Fibrinogen: Controls 298.87 ± 5.18 mg/dL, Migraine 316.12 ± 3.63 mg/dL, MA 318 ± 4.95 mg/dL, MO 313.70 ± 5.34 mg/dL (Migraine vs. control p = 0.007; MA vs. control p = 0.02). hs-CRP: Controls 2.56 ± 0.28 mg/L, Migraine 3.43 ± 0.27 mg/L, MA 3.52 ± 0.38 mg/L, MO 3.30 ± 0.39 mg/L (Migraine vs. control p = | Migraine, particularly migraine with aura, is associated with elevated biomarkers of hypercoagulability (fibrinogen, Factor II) and inflammation (hs-CRP), especially in women. |

| | | | | | | |
|---|---|---|---|---|---|---|
| | | | | | 0.03). Factor II activity: Controls 108.14 ± 1.23%, Migraine 110.21 ± 0.96%, MA 111.07 ± 1.29%, MO 109.17 ± 1.45%. | |

**Table S5. Category 5: Radiological and Neuroimaging Findings (2 studies)**

| Author Year | Study Design | Sample Size (N migraine/N controls) | Migraine Subtype | Assessment Method | Main Findings (Exact Numbers) | Interpretation |
|---|---|---|---|---|---|---|
| **Avci et al., 2015* (WMH findings)** | Case–control. | 216 migraine/216 controls (ages 18–50 years)/total 432. | MO (n = 143), MA (n = 73). | White matter hyperintensities (WMHs) on MRI. | WMHs prevalence: Migraine patients 69/216 (31.9%, 1.68 ± 3.12 mg/dL) vs. Controls 21/216 (9.7%, 0.3 ± 1.3, p < 0.001); OR 4.35 (95% CI 1.90–9.45, p ≤ 0.001). WMH location: Predominantly frontal lobe and subcortical areas. | Significantly higher WMH prevalence and burden in migraine patients. |
| **Vijiaratnam et al., 2016** | Retrospective observational study. | 505 patients with migraine; 248 migraine without aura, 257 migraine with aura (MA). | Migraine with aura (MA), migraine without aura (ICHD-3 criteria for typical migraine aura). | Imaging studies (CT brain, MRI brain, carotid Doppler studies); cardiovascular risk factors (hypertension, hypercholesterolemia, type 2 diabetes mellitus, smoking, family history of stroke, atrial fibrillation); white matter hyperintensities on MRI. | White matter hyperintensities on MRI: MA 39% vs. migraine without aura 41% (p = 0.79). Carotid Doppler studies: 21% of MA patients investigated; only 1 patient had abnormal result. | Patients with migraine with aura and without aura were largely similar in cardiovascular risk profile. Imaging (CT and MRI) in either group had almost no clinical value, with no clinically significant findings. White matter hyperintensities were associated with older age and traditional cardiovascular risk factors rather than migraine subtype. |

*Note: Avci et al., 2015 appears in both Category 4 (hs-CRP findings) and Category 5 (WMH findings) as the study assessed two independent vascular outcomes with no significant correlation.

**Table S6. Category 6: Genetic and Molecular Biomarkers (3 studies)**

| Author Year | Study Design | Sample Size (N migraine/N controls) | Migraine Subtype | Assessment Method | Main Findings (Exact Numbers) | Interpretation |
|---|---|---|---|---|---|---|
| **García-Martín et al., 2019** | Case–control genetic association study. | 283 migraine/287 controls (total N = 570 Caucasian Spanish individuals). | MWA, MwoA. | NOS3 rs2070744 single-nucleotide polymorphism (SNP) genotyping by TaqMan-based qPCR (endothelial nitric oxide synthase gene). | Rs207044: Minor allele OR 0.91 (0.72–1.15), NS). rs2070744CC: Minor allele OR 0.80 (0.52–1.23). | NOS3 rs2070744CC genotypes were significantly more frequent in patients with a family history of migraine. NOS3 rs2070744 SNP is not associated with the risk for migraine. |
| **García-Martín et al., 2015** | Case–control genetic study. | 197 migraine/308 controls (total N = 505). | MWA (n = 98); MO (implied by "presence or absence of aura"); 113 onset <15 years; 147 positive family history. | Neuronal nitric oxide synthase gene (NOS1/nNOS, chromosome 12q24.22) functional SNPs: rs7977109 and rs693534 genotypes and allelic variants by TaqMan-based qPCR. | rs7977109: Minor allele OR 0.94 (95% CI 0.72–1.23, NS). rs693534: Minor allele OR 0.88 (95% CI 0.68–1.15, NS). | NOS1 rs7977109 and rs693534 genotypes/allelic variants NOT associated with migraine risk. |
| **Kuan et al., 2019** | Cross-sectional study. | 1593 migraine patients/Genetic data subset: 351. | Migraine with or without aura. | Specifically, it explored associations between migraine susceptibility loci (genes) and | Genetic associations: rs11172113 in LRP1 was associated with syncope risks: OR of 4.00 (95% | Study found a genetic association with LRP1 and highlighted the significant |

| | | | | | | |
|---|---|---|---|---|---|---|
| | | | | syncope risks. The study also mentioned that LRP1 is important for vascular wall integrity and that allele C of rs11172113 in LRP1 is associated with lower gene expression in human vascular tissues. | CI 1.03–15.45) and a p-value of .045. No significant associations: rs10166942 in TRPM8, rs655484 in DLG2, or rs3781545 in GFRA1. | psychological burden of comorbid syncope, suggesting vascular dysfunction as a potential common underlying mechanism. |

**Table S7. Category 7: Neurocognitive Profiles (2 studies)**

| Author Year | Study Design | Sample Size (N migraine/N controls) | Migraine Subtype | Assessment Method | Main Findings (Exact Numbers) | Interpretation |
|---|---|---|---|---|---|---|
| **George et al., 2020 (ARIC-NCS)** | Prospective cohort (21-year follow-up). | 1397 migraine/1243 severe non-migraine headache/9955 no migraine history (total N = 12,495). | Migraine: headache ≥4 h + throbbing/pounding/pulsating/unilateral + nausea/vomiting/photophobia/phonophobia + ≥1 year history; MWA subtype assessed separately; Severe non-migraine headache: ≥4 h but not all migraine criteria. | Incident dementia adjudicated from ARIC-NCS visits 5 (2011–2013) and 6 (2016–2017) - history of migraine symptoms was associated cross-sectionally with cerebral white matter hyperintensities. | Cumulative dementia incidence: No migraine 18.5%; Severe non-migraine headache 15.8%; Migraine 16.7%. Migraine and incident dementia fully adjusted: HR 1.04 (NS). MWA and dementia fully adjusted: HR 1.12 (NS). Severe non-migraine headache: No association with dementia. No sex interaction: p = NS. | No association between history of migraine and incident dementia in ARIC cohort. |
| **Wen et al., 2016** | Prospective population-based cohort study (Rotterdam Study). | 6709 participants: 1021 definite migraineurs, 288 probable migraineurs/5399 non-migraineurs. | Definite migraine, migraine with aura, migraine without aura, probable migraine, non- migraine (adapted ICHD-II criteria). | General cognitive function (MMSE, g-factor) and domain-specific cognitive tests (Stroop test, verbal fluency, letter-digit substitution, Purdue pegboard); participants aged 45+ years, mean age migraineurs 63.8 ± 11.1 years. | Migraineurs vs. non-migraineurs: MMSE 0.21/global cognition 0.10/women 0.25/men 0.13. Migraineurs with aura vs. non-migraineurs: MMSE 0.39/Global cognition 0.13. | Migraineurs, particularly migraineurs with aura, tend to score higher in cognition tests than non-migraineurs. |

*Full references for all studies included in this table and a full list of abbreviations are provided in the main manuscript's reference and abbreviations lists.*
