# Supplementary material for: Migraine and Cardiovascular Risk: A Scoping Review of Vascular Outcomes, Risk Assessment, and Endothelial Dysfunction
Source: Life (Basel). 2026 May 27;16(6):900. doi: 10.3390/life16060900 (PMC13302511; doi:10.3390/life16060900)
Supplement: Supplementary file 1 [file life-16-00900-s001.zip › Supplementary File S2.pdf]

Supplementary File S2

Table S8. PRISMA-ScR Checklist: Preferred Reporting Items for Systematic Reviews and Meta-Analyses Extension for Scoping Reviews (Tricco et al., 2018).

Section	Item #	PRISMA-ScR Checklist Item	Reported on Page/Section (applied to present review)
**TITLE**	1	**Title** Identify the report as a scoping review.	The title of the manuscript explicitly states: "Migraine and Cardiovascular Risk: A Scoping Review of Vascular Outcomes, Risk Assessment, and Endothelial Dysfunction". The term 'Scoping Review' is included directly in the title, satisfying this item. [OSF Registration—Title field].
**ABSTRACT**	2	**Structured summary** Provide a structured summary that includes (as applicable): background, objectives, eligibility criteria, sources of evidence, charting methods, results and conclusions that relate to the review questions and objectives.	The abstract provides a structured summary covering: (a) background on migraine as a cardiovascular risk marker; (b) objectives—mapping evidence on migraine and cardiovascular/vascular risk across seven thematic categories; (c) eligibility criteria: adults ≥ 18 years, PCC framework, 2015–2025, English, PubMed/MEDLINE; (d) source of evidence PubMed/MEDLINE, March 2026, n = 161 retrieved; (e) charting methods standardized Excel extraction form, two independent reviewers; (f) results: 43 studies included; (g) conclusions: comprehensive evidence map with identification of knowledge gaps. [OSF Registration—Description field].
**INTRODUCTION**	3	**Rationale** Describe the rationale for the review in the context of what is already known. Explain why the review questions/objectives lend themselves to a scoping review approach.	The rationale is described in the Background section of the OSF registration: existing systematic reviews and meta-analyses are restricted to single outcome domains, use inconsistent migraine phenotyping, and present high between-study heterogeneity (Schürks et al., 2009; Adnyana et al., 2022; Zhao et al., 2024). No prior review has mapped the full vascular spectrum. The scoping review approach was selected over a systematic review due to the significant clinical and methodological heterogeneity characterizing the intersection of migraine and cardiovascular risk across multiple research domains simultaneously. [OSF Registration—Background and Type of Review sections].
	4	**Objectives** Provide an explicit statement of the questions and objectives	Primary research question: "What is the current state of evidence on the association between migraine and cardiovascular risk, encompassing

		being addressed with reference to their key elements (e.g., population or participants, concepts, and context) or other relevant key elements used to conceptualize the review questions and/or objectives.	vascular outcomes, endothelial dysfunction, and cardiovascular risk assessment in adult populations (≥18 years), as published in English-language human clinical studies between 2015 and 2025?"<br>Seven secondary research questions address (1) cardiovascular risk factors and scores; (2) stroke risk and cerebrovascular outcomes; (3) structural and functional vascular abnormalities; (4) inflammatory and circulating biomarkers; (5) radiological and neuroimaging findings; (6) genetic and molecular variants; (7) neurocognitive profiles and dementia risk.<br>[OSF Registration—Primary research question(s) and Secondary research question(s) sections].
**METHODS**	5	**Protocol and registration**<br>Indicate whether a review protocol exists; state if and where it can be accessed (e.g., Web address); and if available, provide registration information, including the registration number.	The review protocol is registered on the Open Science Framework (OSF). The registration is retrospective, submitted concurrently with manuscript preparation. At the time of freezing this registration, all review stages—search, screening, extraction, synthesis, and reporting—had been completed. The OSF registration is publicly accessible and was assigned a persistent DOI through the OSF platform.<br>[OSF Registration—Current review stage and Data management and sharing sections].
	6	**Eligibility criteria**<br>Specify characteristics of the sources of evidence used as eligibility criteria (e.g., years considered, language, publication status), and provide the rationale.	Eligibility criteria were defined using the PCC framework:<br>• Population (P): Adults ≥ 18 years diagnosed with any migraine subtype (episodic, chronic, with/without aura) per ICHD or established clinical criteria.<br>• Concept (C): Vascular outcomes, endothelial dysfunction, cardiovascular risk assessment, stroke risk, neuroimaging findings, inflammatory and molecular biomarkers, neurocognitive profiles.<br>• Context (Co): Human clinical studies (cross-sectional, case–control, cohort, RCTs), English language, 2015–2025.<br>Exclusion criteria: pediatric populations (<18 years), pregnancy-related conditions, secondary vascular disorders (CADASIL, Sturge–Weber, arterial dissection), animal/in vitro models, case reports (n < 10), secondary literature, non-English publications, studies without relevant outcomes.<br>Post hoc minimum sample size of 400 participants

		applied. [OSF Registration—Inclusion and exclusion criteria section].
7	**Information sources*** Describe all information sources in the search (e.g., databases with dates of coverage and contact with authors to identify additional sources), as well as the date the most recent search was executed.	A single systematic literature search was conducted exclusively in PubMed/MEDLINE (National Library of Medicine, Bethesda, MD, USA), searched in March 2026, via the native PubMed interface (https://pubmed.ncbi.nlm.nih.gov/). PubMed was selected due to its comprehensive coverage of the core disciplines relevant to this review (neurology, vascular biology, cardiology, and epidemiology). No grey literature, backward/forward citation searching, or author contact was conducted. No additional databases were searched. [OSF Registration—Databases, Interfaces, and Grey Literature Sections].
8	**Search** Present the full electronic search strategy for at least one database, including any limits used, such that it could be repeated.	The full Boolean query string is provided in the OSF registration (Query strings section). The strategy comprised three blocks: Block 1—migraine terms restricted to [Title] field (9 terms including migraine, chronic migraine, episodic migraine, hemiplegic migraine); Block 2—vascular/endothelial terms in [Title/Abstract] (17 thematic sub-blocks covering FMD, cIMT, PWV, vWF, ET-1, VEGF, ADMA, eNOS, EPCs, adhesion molecules, PTX3, atherosclerosis, cerebrovascular reactivity, and others); Block 3—NOT exclusion filters (pregnancy, pediatric, animal, CADASIL, arterial dissection terms). PubMed filters applied: 2015–2025, English, abstract available, human species, adults only. Total retrieved: 161 records. [OSF Registration—Query strings and Search strategy justification sections].
9	**Selection of sources of evidence†** State the process for selecting sources of evidence (i.e., screening and eligibility) included in the scoping review.	Two-stage screening process: Stage 1—Title and abstract screening of all 161 records against PCC eligibility criteria, conducted independently by two human reviewers. Stage 2—Full-text screening of records passing Stage 1, with additional application of the ≥400 participant threshold (n = 104 excluded), exclusion of reviews/meta-analyses not identified at Stage 1 (n = 3), exclusion of studies without relevant outcomes (n = 10), and exclusion for full-text inaccessibility (n = 1). Discrepancies were resolved through discussion; the third reviewer consulted when consensus was not reached. Final included

			studies: n = 43. [OSF Registration—Screening stages, Screener instructions, and Screening reliability sections].
	10	**Data charting process†** Describe the methods of charting data from the included sources of evidence (e.g., calibrated forms or forms that have been tested by the team before their use, and whether data charting was done independently or in duplicate) and any processes for obtaining and confirming data from investigators.	Data extraction was conducted in two sequential stages using a standardized digital extraction form (Appendix A: Table A1–7). Stage 1: One primary human reviewer systematically extracted all predefined entities from the full text of each of the 43 included studies. Stage 2: A second human reviewer independently verified all extracted data against original publications. Discrepancies were resolved through discussion with the third reviewer and arbitration. No AI-assisted extraction was employed. No author contact was undertaken to obtain missing data. [OSF Registration—Extraction stages, Extractor instructions, and Extraction reliability sections].
	11	**Data items** List and define all variables for which data were sought and any assumptions and simplifications made.	Extracted entities included: (1) Metadata: author/year, country, funding; (2) Study design: design type, follow-up duration, data source; (3) Sample characteristics: total N, N migraine, N controls, age, sex, ethnicity; (4) Independent variable: migraine subtype (MA, MO, CM, EM), diagnostic criteria (ICHD-II/III, ICD codes), migraine characteristics (onset age, frequency, duration, aura frequency); (5) Dependent variables across seven thematic categories (cardiovascular risk factors/scores, stroke outcomes, vascular assessment measures, biomarkers, neuroimaging findings, genetic SNPs, cognitive outcomes); (6) Effect size estimates: OR, HR, RR, means ± SD, p-values, 95% CI; (7) Authors' interpretation and direction of association; (8) Thematic category assignment (primary and secondary where applicable). [OSF Registration—Entities to extract section].
	12	**Critical appraisal of individual sources of evidence (optional)** If done, provide a rationale for conducting a critical appraisal of included sources of evidence; describe the methods used and how this information was used in any data synthesis (if appropriate).	No formal quality assessment or risk of bias evaluation was conducted. This decision is consistent with the scoping review methodology adopted (Arksey & O'Malley, 2005; Levac et al., 2010), which explicitly does not require critical appraisal of individual studies. No tools such as the Cochrane Risk of Bias tool, GRADE, ROBINS-I, or Newcastle–Ottawa Scale were applied. All included studies meeting eligibility criteria were included in the narrative synthesis regardless of methodological quality, with study design and

			sample size reported transparently. [OSF Registration—Quality assessment section].
	13	**Summary measures** Not applicable for scoping reviews.	Not applicable. This is a scoping review; no meta-analytic pooling or summary effect measures were calculated. Narrative synthesis was employed throughout. [OSF Registration—Planned data transformations and Synthesis plan sections].
	14	**Synthesis of results** Describe the methods of handling and summarizing the data that were charted.	Narrative synthesis was conducted across all seven thematic categories without meta-analytic pooling, consistent with Arksey & O'Malley (2005) and Levac et al. (2010). Synthesis steps: (1) Thematic organization of 43 studies into seven predefined categories; (2) Within-category narrative synthesis identifying convergent findings, divergent findings, direction of association, and subgroup patterns (MA vs. MO vs. CM); (3) Cross-category synthesis identifying overarching patterns and paradoxes (e.g., 'healthy vascular system' paradox, cardioembolic stroke pathway, 'migraine-cognition paradox'); (4) Knowledge gap identification based on limited evidence categories and methodological inconsistencies; (5) Reporting in accordance with PRISMA-ScR guidelines. [OSF Registration—Synthesis plan section].
	15	**Risk of bias across studies** Not applicable for scoping reviews.	Not applicable. Consistent with scoping review methodology, no assessment of publication bias or risk of bias across studies was conducted. No funnel plots, Egger's test, or similar procedures were applied. The potential for publication bias is acknowledged as a limitation. [OSF Registration—Publication bias analyses and Quality assessment sections].
	16	**Additional analyses** Not applicable for scoping reviews.	Not applicable. No sensitivity analyses, subgroup analyses, or meta-regression were conducted, consistent with the narrative synthesis approach adopted. [OSF Registration—Sensitivity analyses/robustness checks section].
**RESULTS**	17	**Selection of sources of evidence** Give numbers of sources of evidence screened, assessed for eligibility, and included in the review, with reasons for exclusions at each stage,	Records identified via PubMed/MEDLINE: n = 161. No duplicates (single database). Records assessed at full-text: subset of Stage 1 survivors. Exclusions at full-text stage: n = 104 (sample size < 400 participants); n = 3 (reviews/meta-analyses not identified at Stage 1); n = 10 (≥400 participants but no relevant outcomes); n = 1 (full text inaccessible).

		ideally using a flow diagram.	Records initially meeting all criteria: n = 44. Final included studies after post-screening exclusion: n = 43. A PRISMA-ScR flow diagram documenting this process is included in the manuscript. [OSF Registration—Outcome of Screening (Screening stages section) and PRISMA flow diagram].
	18	**Characteristics of sources of evidence** For each source of evidence, present the characteristics for which data were charted and provide the citations.	Characteristics of all 43 included studies are presented in Appendix A, Tables A1–7, organized across seven thematic categories. For each study, the following characteristics are reported: author/year, country/cohort, study design (cross-sectional, case–control, prospective/retrospective cohort, RCT, population-based cohort, twin study), total sample size (N ranging from 417 to 1,195,696), N migraine, N controls, age range/mean, sex distribution, migraine subtype (MA, MO, CM, EM), diagnostic criteria (ICHD-II/III, ICD codes, clinical diagnosis, self-report), assessment method, main findings, and thematic category assignment. Collectively, the included studies encompassed over 1,500,000 participants. [OSF Registration—Entities to extract section; Appendix A, Tables A1–7].
	19	**Critical appraisal within sources of evidence (optional)** If done, present data on critical appraisal of included sources of evidence (see item 12).	Not conducted. As stated under item 12, no formal quality assessment or risk of bias evaluation was performed for any included source of evidence. This is consistent with the scoping review methodology adopted and is acknowledged as a limitation in the manuscript. [OSF Registration—Quality assessment section].
	20	**Results of individual sources of evidence** For each included source of evidence, present the relevant data that were charted that relate to the review questions and objectives.	Results for each of the 43 studies included are presented in Appendix A, Tables A1–7, organized across the seven thematic categories. For each study, the following are reported: exact numerical results (OR, HR, RR, means ± SD, p-values, 95% CI), both unadjusted and multivariable-adjusted estimates where available, and the authors' interpretation of the direction of association (positive, inverse, neutral/non-significant). Non-significant findings were extracted and reported with equal rigor as statistically significant results. [OSF Registration—Entities to extract and Extractor instructions sections; Appendix A, Tables A1–7].
	21	**Synthesis of results** Summarize and/or present the	Narrative synthesis results are presented across the seven thematic categories in the Results section

		charting results as they relate to the review questions and objectives.	of the manuscript: Category 1—Cardiovascular Risk Assessment (n studies); Category 2—Stroke Risk and Outcomes; Category 3—Structural and Functional Vascular Assessment; Category 4—Inflammatory and Circulating Biomarkers (n = 2 studies); Category 5—Radiological and Neuroimaging Findings (n = 2 studies); Category 6—Genetic and Molecular Biomarkers; Category 7—Neurocognitive Profiles (n = 2 studies). Cross-category synthesis identifies overarching patterns including the 'healthy vascular system' paradox (Categories 1 and 3), the cardioembolic stroke pathway (Categories 1 and 2), and the 'migraine-cognition paradox' (Categories 5 and 7). [OSF Registration—Synthesis plan section; manuscript Results section].
	22	**Risk of bias across studies** Not applicable for scoping reviews.	Not applicable. [OSF Registration—Publication bias analyses section].
	23	**Additional analyses** Not applicable for scoping reviews.	Not applicable. [OSF Registration—Sensitivity analyses / robustness checks section].
**DISCUSSION**	24	**Summary of evidence** Summarize the main results (including an overview of concepts, themes, and types of evidence available), link to the review questions and objectives, and consider the relevance to key groups.	The main findings are organized across seven thematic categories and summarized in the Discussion section of the manuscript. Key themes include: (1) migraine shows an inverse/neutral association with traditional cardiovascular risk factors ('healthy vascular system' paradox); (2) migraine with aura is the primary driver of ischemic stroke risk via cardioembolic rather than atherosclerotic pathways (PFO prevalence up to 79%); (3) no significant macrovascular structural damage (PWV, CAC, cIMT); (4) elevated inflammatory biomarkers (hs-CRP, fibrinogen, vWF) in migraine patients; (5) higher WMH burden on MRI; (6) non-significant associations for NOS gene polymorphisms; (7) no increased dementia risk despite higher WMH burden ('migraine-cognition paradox'). [OSF Registration—Expectations/hypotheses and Synthesis plan sections; manuscript Discussion section].
	25	**Limitations** Discuss the limitations of the scoping review process.	Limitations acknowledged in the OSF registration and manuscript include: (1) single database search (PubMed/MEDLINE only)—potential for missed relevant studies in other databases; (2) no grey literature search; (3) no formal inter-rater

			reliability testing (Cohen's kappa not calculated); (4) no quality assessment or risk of bias evaluation of included studies; (5) no retraction status check via retraction databases; (6) post hoc application of the ≥400 participant threshold; (7) retrospective registration; (8) limited evidence base in Categories 4, 5, and 7 (n = 2 studies each); (9) significant clinical and methodological heterogeneity across included studies limiting cross-study comparisons. [OSF Registration—Current review stage, Quality assessment, and Miscellaneous screening details sections].
	26	**Conclusions** Provide a general interpretation of the results with respect to the review questions and objectives, as well as potential implications and/or next steps.	The conclusions, detailed in the manuscript, address the five expected outcomes stated in the OSF registration: (1) a comprehensive evidence map linking migraine to cardiovascular and vascular risk across seven domains; (2) identification of the most studied vascular assessment methods and biomarkers in migraine populations; (3) clarification of the MA-specific cardioembolic stroke pathway; (4) identification of critical knowledge gaps and methodological inconsistencies; (5) a foundation for future hypothesis-driven systematic reviews or clinical risk stratification protocols in migraine patients. [OSF Registration—Description (Expected outcomes) and Expectations/hypotheses sections].
**FUNDING**	27	**Funding** Describe sources of funding for the included sources of evidence, as well as sources of funding for the scoping review. Describe the role of the funders of the scoping review.	This scoping review received no external funding. The work was conducted independently without financial support from any public, commercial, or not-for-profit funding agency. The authors declare no conflicts of interest. No potential conflicts of interest exist in relation to the design, conduct, reporting, or outcomes of this scoping review. [OSF Registration—Funding and Conflicts of interest sections].

** Where sources of evidence are compiled from bibliographic databases, social media platforms, and websites.*
† A more inclusive/heterogeneous term used to account for different types of evidence or data sources.
‡ Critical appraisal (Items 12 and 19) is optional for scoping reviews.
Items 13, 15, 16, 22, and 23 are listed as 'Not applicable for scoping reviews' per Tricco et al. (2018).
Reference: Tricco AC, Lillie E, Zarin W, et al. PRISMA Extension for Scoping Reviews (PRISMA-ScR): Checklist and Explanation. Ann Intern Med. 2018;169(7):467–473. doi:10.7326/M18-0850
OSF Registration: Cuciureanu, D. I., Nadejde, A., Vulpoi, G., Bistriceanu, C. E., Antochi, F., & Roceanu, A. (2026, April 3). Migraine and Cardiovascular Risk: A Scoping Review of Vascular Outcomes, Risk Assessment, and Endothelial Dysfunction. https://doi.org/10.17605/OSF.IO/YV95Z

Search strategy: The full PubMed search query, accessed in March 2026, integrated with Boolean operators and study filters, is detailed below:

("migraine"[Title] OR "migraines"[Title] OR "migraine disorder"[Title] OR "migraine disorders"[Title] OR "migrainous"[Title] OR "migraine headache"[Title] OR "chronic migraine"[Title] OR "episodic migraine"[Title] OR "hemiplegic migraine"[Title] OR "familial hemiplegic migraine"[Title]) AND (("endothelial dysfunction"[Title/Abstract] OR "endothelial function"[Title/Abstract] OR "endothelial impairment"[Title/Abstract] OR "endothelial damage"[Title/Abstract] OR "endothelial activation"[Title/Abstract] OR "endothelial injury"[Title/Abstract] OR "endothelium"[Title/Abstract] OR "vascular endothelium"[Title/Abstract] OR "endothelial cell"[Title/Abstract] OR "endothelial cells"[Title/Abstract]) OR ("vascular dysfunction"[Title/Abstract] OR "vascular function"[Title/Abstract] OR "vascular impairment"[Title/Abstract] OR "cerebrovascular dysfunction"[Title/Abstract] OR "vascular damage"[Title/Abstract]) OR ("cardiovascular risk"[Title/Abstract] OR "vascular risk"[Title/Abstract] OR "atherosclerosis"[Title/Abstract] OR "atherosclerotic"[Title/Abstract] OR "subclinical atherosclerosis"[Title/Abstract]) OR ("flow-mediated dilation"[Title/Abstract] OR "flow mediated dilatation"[Title/Abstract] OR "FMD"[Title/Abstract] OR "flow-mediated vasodilation"[Title/Abstract] OR "brachial artery reactivity"[Title/Abstract]) OR ("endothelial progenitor cell"[Title/Abstract] OR "endothelial progenitor cells"[Title/Abstract] OR "EPC"[Title/Abstract] OR "EPCs"[Title/Abstract] OR "circulating endothelial cells"[Title/Abstract]) OR ("von Willebrand factor"[Title/Abstract] OR "vWF"[Title/Abstract] OR "VWF"[Title/Abstract] OR "von Willebrand"[Title/Abstract]) OR ("endothelin"[Title/Abstract] OR "endothelin-1"[Title/Abstract] OR "ET-1"[Title/Abstract]) OR ("VEGF"[Title/Abstract] OR "vascular endothelial growth factor"[Title/Abstract]) OR ("carotid intima-media thickness"[Title/Abstract] OR "carotid IMT"[Title/Abstract] OR "cIMT"[Title/Abstract] OR "intima media thickness"[Title/Abstract] OR "intima-media"[Title/Abstract]) OR ("arterial stiffness"[Title/Abstract] OR "pulse wave velocity"[Title/Abstract] OR "PWV"[Title/Abstract] OR "aortic stiffness"[Title/Abstract]) OR ("ADMA"[Title/Abstract] OR "asymmetric dimethylarginine"[Title/Abstract]) OR ("nitric oxide"[Title/Abstract] OR "endothelial nitric oxide"[Title/Abstract] OR "eNOS"[Title/Abstract]) OR ("vascular biomarker"[Title/Abstract] OR "vascular biomarkers"[Title/Abstract] OR "endothelial biomarker"[Title/Abstract] OR "endothelial biomarkers"[Title/Abstract]) OR ("pentraxin"[Title/Abstract] OR "PTX3"[Title/Abstract]) OR ("adhesion molecules"[Title/Abstract] OR "ICAM"[Title/Abstract] OR "VCAM"[Title/Abstract] OR "E-selectin"[Title/Abstract] OR "P-selectin"[Title/Abstract] OR "cell adhesion"[Title/Abstract]) OR ("cerebrovascular reactivity"[Title/Abstract] OR "vasoreactivity"[Title/Abstract] OR "cerebral vasoreactivity"[Title/Abstract])) NOT ("pregnancy"[Title/Abstract] OR "pregnant"[Title/Abstract] OR "preeclampsia"[Title/Abstract] OR "eclampsia"[Title/Abstract] OR "puerperium"[Title/Abstract] OR "obstetric"[Title/Abstract] OR "gestational"[Title/Abstract] OR "maternal"[Title/Abstract] OR "pediatric"[Title/Abstract] OR "paediatric"[Title/Abstract] OR "children"[Title/Abstract] OR "child"[Title/Abstract] OR "adolescent"[Title/Abstract] OR "adolescents"[Title/Abstract] OR "infant"[Title/Abstract] OR "infants"[Title/Abstract] OR "neonatal"[Title/Abstract] OR "newborn"[Title/Abstract] OR "Sturge-Weber"[Title/Abstract] OR "Sturge Weber"[Title/Abstract] OR "arterial dissection"[Title/Abstract] OR "artery dissection"[Title/Abstract] OR "cervical artery dissection"[Title/Abstract] OR "carotid dissection"[Title/Abstract] OR "vertebral dissection"[Title/Abstract] OR "CADASIL"[Title/Abstract] OR "cerebral autosomal dominant arteriopathy"[Title/Abstract] OR "animal model"[Title/Abstract] OR "rat model"[Title/Abstract] OR "mouse model"[Title/Abstract] OR "mice"[Title/Abstract] OR "rats"[Title/Abstract])

Filters applied: Publication years: 2015–2025; language: English; species: human; age: adult (19+); text availability: abstract.
